# The Immune Response in Two Models of Traumatic Injury of the Immature Brain

**DOI:** 10.3390/cells13191612

**Published:** 2024-09-26

**Authors:** Zahra F. Al-Khateeb, Siân M. Henson, Jordi L. Tremoleda, Adina T. Michael-Titus

**Affiliations:** 1 Centre for Neuroscience, Surgery and Trauma, The Blizard Institute, Barts and The London School of Medicine and Dentistry, Queen Mary University of London, London EC1M 6BQ, UK; 2 Translational Medicine and Therapeutics, William Harvey Research Institute, Barts and The London School of Medicine and Dentistry, Queen Mary University of London, London EC1M 6BQ, UK

**Keywords:** traumatic brain injury, neuroinflammation, peripheral immune response, adaptive immunity, innate immunity, controlled cortical impact, repetitive mild traumatic brain injury, juvenile, mice

## Abstract

Traumatic brain injury (TBI) can cause major disability and increases the risk of neurodegeneration. Post-TBI, there is infiltration of peripheral myeloid and lymphoid cells; there is limited information on the peripheral immune response post-TBI in the immature brain—where injury may interfere with neurodevelopment. We carried out two injury types in juvenile mice: invasive TBI with a controlled cortical impact (CCI) and repetitive mild TBI (rmTBI) using weight drop injury and analysed the response at 5- and 35-days post-injury. In the two models, we detected the brain infiltration of immune cells (e.g., neutrophils, monocytes, dendritic cells, CD4+ T cells, and NK cells). There were increases in macrophages, neutrophils, and dendritic cells in the spleen, increases in dendritic cells in blood, and increases in CD8+ T cells and B cells in lymph nodes. These results indicate a complex peripheral immune response post-TBI in the immature brain, with differences between an invasive injury and a repetitive mild injury.

## 1. Introduction

Traumatic brain injury (TBI) can have a major impact on individuals and is associated with significant public health costs. Brain tissue can be damaged by a powerful external impact (closed head injury), a penetrating object (invasive head injury), or a blast wave resulting from an explosion [1,2], and this can lead to complications such as physical disability, neurocognitive impairment, and neuropsychological problems, which may last a lifetime. These complications can occur even after mild injuries [3] and may become apparent several years after injury [4]. Emerging evidence indicates that the mature adult brain and the immature brain respond differently to an injury, despite sharing similar pathophysiological mechanisms, and this is due to the immature brain being at a different developmental phase [5]. Patients who acquired injuries in their childhood or adolescence can present long-term behavioural deficits [6,7,8].

After moderate to severe brain injuries, children struggle more with long-term impairments in cognition and social abilities [9], and similar findings were also reported in animal models [10,11]. Infants and toddlers had a higher mortality rate after an injury than the other age groups [5,12]. The persistent impairments affect their academic performance and social abilities more than in children who sustained an injury at an older age [13,14]. Two main models explain the outcome of TBI in paediatric injury: the first model is “early vulnerability” and the second is “early plasticity”. The first model hypothesises that, as the immature brain is undergoing maturation, the injury hinders this process. The consequences of such injury would not be noticed until later in development [7]. As for the second model, the plasticity of brain tissue would restore the neuronal connections at the cellular level, but this would not be necessarily reflected functionally. This could be related to the insufficient neural network recovery after injury [15]. Extrinsic factors can influence the outcome, such as the nature of the injury, the environment before and after the injury, and pre-existing medical conditions [16].

A key event with a significant impact on the injury outcome is the immune system activation following trauma and the subsequent persistence of neuroinflammation [2]. Post-TBI inflammation is triggered by various factors, e.g., cellular debris, oxidative stress, and the complement system. Damaged neurons and endothelial cells secrete chemokines to recruit local immune cells, as well as peripheral immune cells to the area of injury. While inflammatory reactions help clear cellular debris and could support tissue recovery, chronic activation leads to further tissue damage and neuronal cell death [17]. After TBI, different components of the innate immune system are activated—microglia [18] and astrocytes [19]—but also neutrophil recruitment from the periphery [20], and peripheral monocyte infiltration [21]. There is an increase in pro-inflammatory cytokine production [22]. Chronic activation involves additional components, including CD8+ T cells, CD4+ helper T cells [23], B cells [24] and the complement system [25], which all contribute to autoimmunity.

The immune system response varies depending on the nature and severity of the injury. A focal injury induces the recruitment of neutrophils, which peaks within the first 1 to 2 days after injury, and, later on, other immune cells infiltrate the brain: monocytes, followed later by T cells and B cells [26]. In diffuse injury, the early cellular response is mainly mediated by microglia and astrocytes and is generally prominent in white matter. Some studies reported minimum infiltration or no infiltration of neutrophils, but the infiltration of monocytes peaks 3 days after injury [27]. At the later phase after injury, helper T cells infiltrate the brain, which exacerbates the inflammatory response [27,28].

Only a few studies have analysed the effect of TBI on peripheral lymphoid organs. Immune cells residing in lymphoid organs are affected by the circulating inflammatory mediators that are released after injury [29]. For example, resident macrophages in the spleen respond to these signals by releasing pro-inflammatory cytokines such as TNF-α and IL-1β into the blood [30].

In the immature brain, the immune system plays a crucial role in normal brain development. Any injury that triggers an inflammatory response can lead to immune dysregulation, with age-dependent consequences for brain plasticity and repair [31]. A study investigating TBI in juvenile murine brains found that TBI at P21 results in a greater and more prolonged infiltration of GR-1 granulocytes, mainly neutrophils, and CD45+ leukocytes, lasting up to two weeks post-injury. This differs from adult brains, where such infiltration is limited to three days, highlighting the significant role of neutrophils in TBI within the immature brain [32]. However, there are no studies that have investigated the effect of TBI on other types of immune cells or the peripheral lymphoid organs in juvenile mice.

In a previous study, we investigated neuroinflammation in the immature mouse brain after brain injury [33], at a stage of development proposed to represent the pre-teen level in humans [34]. We used two models that mirrored two types of TBI commonly seen in paediatric and adolescent populations. For invasive injuries, we used the controlled cortical impact (CCI), a widely recognised model for inducing focal brain injuries. To induce a repeated mild TBI (rmTBI), which reproduces repeated concussion, we used the weight drop injury model (WDI) proposed by Bittigau et al. (1998) [35]. This model mimics forces experienced in sports-related injuries, i.e., the acceleration–deceleration effects associated with diffuse injuries. After these injuries in the juvenile brain, the analysis of astrocytes and microglia indicated a mild inflammatory reaction after rmTBI and a more intense inflammatory response after CCI [33].

In the present study, we continued the investigation of the response to injury in these models. We assessed the peripheral immune system response to TBI using two panels of immune markers, in the brain and peripheral immune tissues (blood, spleen, and lymph nodes). We assessed changes at two times post-injury, 5 days and 35 days, considered the acute and subacute stages, respectively, to detect the injury progression. We used flow cytometry and also measured cytokines in brain tissue. We examined the presence of infiltrating immune cells from the innate and adaptive immune system: peripheral macrophages, monocytes, neutrophils, dendritic cells, B cells, T cells, CD4+ T cells, CD8+ T cells, cytotoxic T cells, and natural killer T cells. We analysed other lymphatic tissues to define the patterns of immune cell migration between the periphery and the brain in response to injury. We compared the response to injury to control groups (anaesthesia or craniotomy surgery only) and also to naïve animals.

## 2. Materials and Methods

### 2.1. Animals

Juvenile male CD1 mice, aged 4–5 weeks and weighing between 25–29 g at the start of the study, were obtained from Charles River UK Ltd. (Margate, UK). The mice were housed in groups of three within standard ventilated cages (Allentown Europe, Reading, UK) in a controlled environment maintained at a temperature of 21 ± 1 °C, with a relative humidity of 55% ± 10%. The light cycle was regulated to 12 h of light (from 06:00 to 18:00), followed by 12 h of darkness (from 18:00 to 06:00). The social groups of the mice were kept consistent throughout the duration of the study. The mice had continuous access to water and standard chow (Labdiet^®^ EURodent 14% Diet 5LF2, Labdiet, Brentwood, MO, USA), and were provided with environmental enrichment materials such as nesting and tunnels. All procedures involving animals were approved by the Animal Welfare and Ethical Review Body at Queen Mary University of London and were conducted in accordance with the UK Animals (Scientific Procedures) Act 1986 and related guidelines.

### 2.2. Controlled Cortical Impact (CCI)

**Figure 1:**
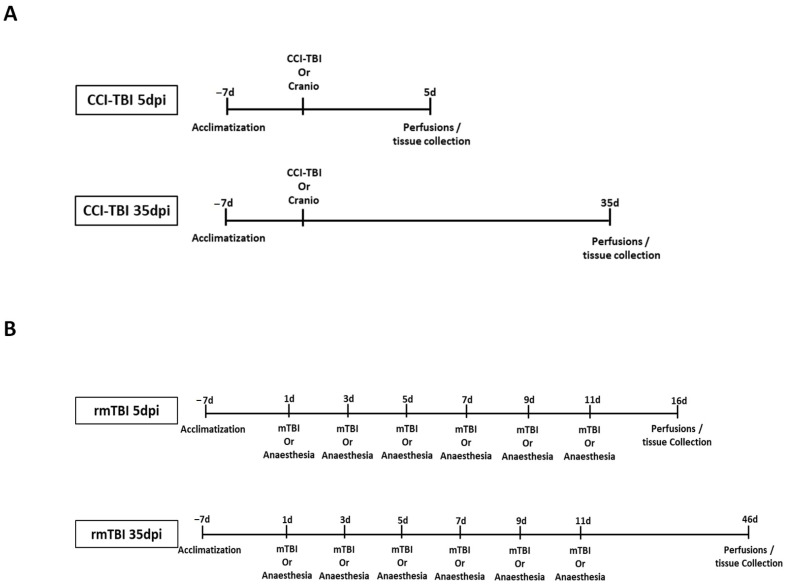
Study timeline of TBI experiments. (**A**) In the CCI experiment, a moderate invasive TBI was induced using the controlled cortical impact (CCI) model. Mice underwent anaesthesia followed by a right lateral craniotomy. Control groups included a craniotomy-only group and a naïve group with no surgical intervention. (**B**) In the rmTBI experiment, the weight drop injury (WDI) model replicated repetitive mild TBI with an intact skull. Mice were briefly anaesthetised and positioned under a PVC tube, where an 80 g weight was dropped from a height of 40 cm onto the head. This procedure was repeated six times with 48 h intervals. Control groups included a repeated anaesthesia group (rSham) and a naïve group with no intervention.

A moderate invasive TBI was induced using the controlled cortical impact (CCI) model. Mice were anaesthetised with an intraperitoneal injection of ketamine (50 mg/kg; Narketan, Vetoquinol, Towcester, UK) and medetomidine (0.5 mg/kg; Domitor, OrionPharma, Reading, UK) in saline (5 mL/kg). A right lateral craniotomy, 3.5 mm in diameter, was performed, after which the injury was delivered using a 3 mm impactor tip at a speed of 3 m/s, with an impact depth of 2.2 mm and a dwell time of 100 milliseconds. This was accomplished using the PCI3000 Precision Cortical Impactor™ (Hatteras Instruments, Inc., Cary, NC, USA) at coordinates 2.0 mm posterior to bregma and 2.5 mm lateral to the midline. Post-impact, the skull flap was repositioned but left unsecured, to accommodate potential swelling, and the skin was sutured closed. All procedures were performed under aseptic conditions. Analgesia was provided pre-emptively and postoperatively, with buprenorphine (0.05 mg/kg, subcutaneous; Vetergesic, Ceva Animal Health Ltd., High Wycombe, UK), every 12 h for up to three days following surgery. The control group underwent a craniotomy without impact, and a separate group of naïve animals received no surgical intervention. The study timeline is illustrated in Figure 1A.

### 2.3. Repetitive Mild Traumatic Brain Injury (rmTBI)

The weight drop injury (WDI) model was used to replicate a repetitive mild TBI with the skull remaining intact (closed skull rmTBI). Mice were briefly anaesthetised with 4% isoflurane in oxygen for 2 min before being positioned on tissue paper beneath a vertical PVC tube, with a foam cushion placed 10 cm below. An 80 g weight was then released from a height of 40 cm, targeting the midline of the head. The impact modelled acceleration–deceleration forces as the mice fell through the tissue paper onto the foam cushion. This procedure was repeated five more times, with a 48 h interval between impacts, totalling six impacts. A sham group was subjected to repeated anaesthesia only (rSham), while a naïve group received no intervention. The timeline of the study is illustrated in Figure 1B.

### 2.4. Tissue Sample Collection

Animals underwent terminal anaesthesia, and blood was collected by cardiac puncture prior to decapitation. Brain tissue was immediately dissected, and one hemisphere was snap-frozen in liquid nitrogen and then stored at −80 °C for cytokine analysis. The other half was placed in cold phosphate-buffered saline (PBS) for flow cytometry analysis. In the CCI group, there was separate analysis of the ipsilateral and contralateral hemispheres. Other lymphatic tissue such as the spleen, upper lymph nodes (ULN, axillary and cervical), and lower lymph nodes (LLN, inguinal) were placed in cold PBS for flow cytometry analysis preparation. Blood samples were collected in lithium heparin tubes and subsequently centrifuged at 10,000× *g* to separate plasma and isolate immune cells.

### 2.5. Flow Cytometry Analysis

We designed two panels to explore innate (Table 1) and adaptive (Table 2) immune cells with flow cytometry. The innate panel assessed total leukocytes infiltrating the brain (CD45+), peripheral macrophages (CD45+ and F4/80), monocytes (CD45+, CD11b+, Ly6C+, and Ly6G-), neutrophils (CD45+, CD11b+, and Ly6G+), and dendritic cells (CD45+, CD11c+, and MHCII+). The adaptive panel assessed B cells (CD45+ and CD45R+), total T cells (CD45+ and CD3+), CD4+ T cells (CD45+, CD3+, and CD4+), CD8+ T cells (CD45+, CD3+, and CD8a+), cytotoxic T cells (CD45+, CD3+, and CD335+), and natural killer cells (CD45+, CD3-, and CD335+).

The preparation of the brain hemispheres involved the digestion of tissue using Accutase (Merck Millipore, Dorset, UK) at room temperature, then dispersion of cells mechanically by pipetting. Samples were washed then treated with 0.9 M sucrose by centrifuging at 800× *g* for 10 min at 4 °C. This was followed by washing and resuspension in PBS. For blood sample preparation, immune cells were collected using Histopaque^®^1077 solution (Sigma-Aldrich, Saint Louis, MO, USA) according to manufacturer’s instructions. For spleen and lymph node processing, each sample was cut into fine pieces on a 70 μm mesh (Greiner Bio-One, Frickenhausen, Germany) using sterile scissors. Cells were extracted by passing cold PBS through the mesh. Next, extracted cells were washed and resuspended in PBS for staining. All prepared samples (except lymph nodes) were split into two sets, with each set being stained using a different panel. Cells were incubated in the dark for 15 min, followed by washing in PBS. Last, 2% paraformaldehyde was used to resuspend and fix cells.

**Figure 2:**
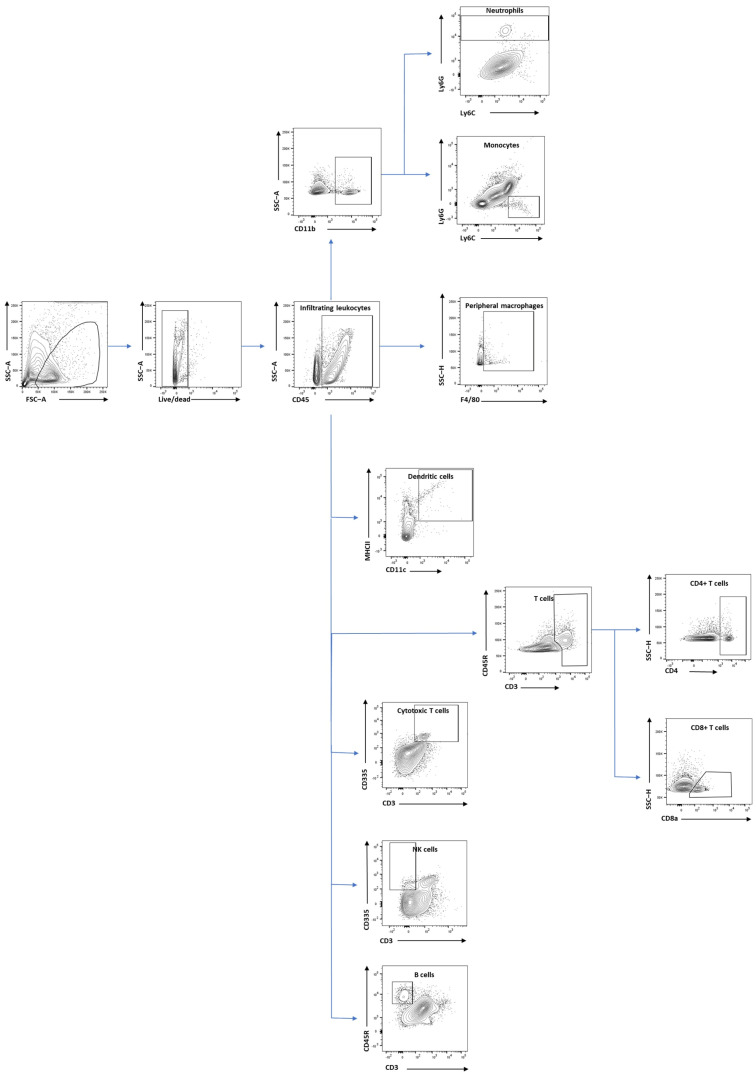
Immune cells gating in brain tissue. Gating of different infiltrating immune cells including neutrophils, macrophages, monocytes, dendritic cells, the total population of T cells, CD8+ T cells, CD4+ T cells, cytotoxic T cells, NK cells, and B cells.

**Figure 3:**
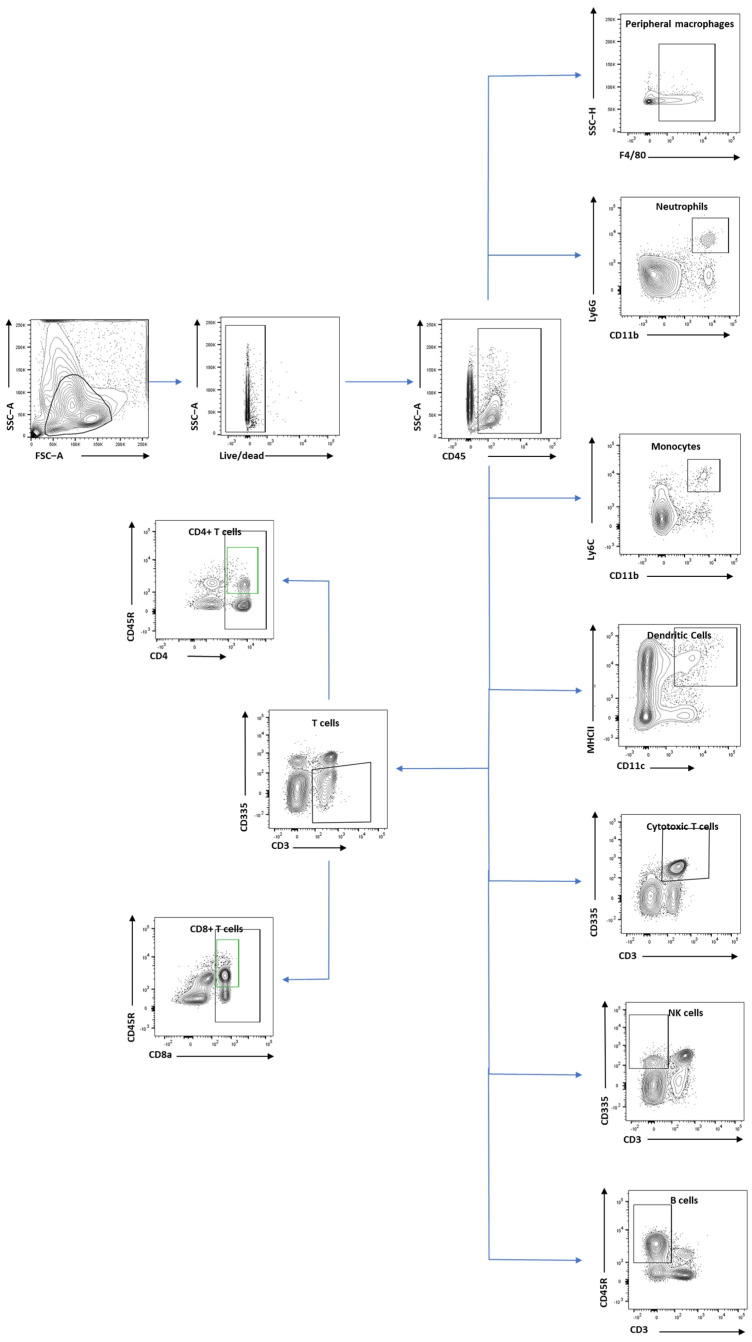
Immune cells gating in spleen, blood and lymph nodes. Gating of different immune cells including neutrophils, macrophages, monocytes, dendritic cells, the total population of T cells, CD8+ T cells, CD4+ T cells, cytotoxic T cells, NK cells, and B cells.

Controls were prepared for each panel before running the flow cytometry analysis. These included unstained cells, control beads stained with a single marker, and cells heat-treated for 10 min and then stained with Live/Dead dye (Zombie NIR, BioLegend UK Ltd., London, UK). All samples were analysed using an LSR Fortessa (BD Life Sciences, Franklin Lakes, NJ, USA) equipped with four lasers: 488 nm blue laser, 405 nm violet laser, 641 nm red laser, and 561 nm yellow-green laser. Data were analysed using FlowJo software version 10.7.1 (BD Life Sciences, Franklin Lakes, NJ, USA). The gating of cells extracted from brain is shown in Figure 2, and Figure 3 shows the gating of spleen, blood, and lymph nodes samples.

### 2.6. Cytokine Array

Cytokine levels were measured using the V-PLEX proinflammatory panel 1 mouse kit, catalogue No. K15048D-1 (Meso Scale Diagnostics (MSD), Rockville, MD, USA). Samples were prepared and plates were used according to manufacturer’s instructions. Plates were analysed using MESO QuickPlex SQ 120MM (MSD, Rahway, NJ, USA). The cytokines analysed were IFN-γ, IL-1β, IL-2, IL-4, IL-5, IL-6, IL-10, IL-12p70, KC/GRO (CXCL1), and TNF-α. For sample preparation, 25 mg snap-frozen brain tissue was used, and the tissue was cut into small pieces on ice. The tissue fragments were suspended in lysate buffer (RIPA Buffer containing protease/phosphatase inhibitors) and then crushed with Pellet Pestle^®^ (Sigma-Aldrich, London, UK). After that, samples were placed on rotating wheels and incubated for 20 min at 4 °C. Next, samples were sonicated 3 times, 15 to 20 sec each, followed by centrifugation for 20 min at 10,000× *g* (4 °C). The supernatant was collected and aliquoted; some aliquots were stored at −80 °C for future work and the remaining aliquots were used for the bicinchoninic acid (BCA) assay to measure protein concentration and, subsequently, cytokine concentrations. For the protein concentration, the Pierce BCA protein assay kit (Invitrogen, Life Technologies Ltd., Paisley, UK) was used. A standard curve was prepared according to the manufacturer’s instructions. Samples were diluted with MSD solution, Diluent 41, to a concentration of 2.5 mg/µL, were added to the plate in duplicate and processed according to manufacturer’s instructions. Plates were read using the MSD instrument and the software Discovery Workbench^®^ 4.0 (MSD, Rahway, NJ, USA) was used to generate standard curves for each cytokine and calculate the concentrations in samples.

### 2.7. Statistical Analysis

GraphPad Prism software (version 9; GraphPad Software Inc., La Jolla, CA, USA) was used for statistical analysis. Data were plotted as median—minimum to maximum, showing all experimental points. Outliers were identified and removed using the ROUT method. The differences between the naïve, the sham, and the injured groups were compared using Friedman’s test (two-way analysis of variance by ranks) followed by post hoc tests for unequal sample sizes, the Dunn post hoc test, and the significance level was set at *p* < 0.05. Data will be made available upon request.

## 3. Results

### 3.1. Immune System Response in Brain Tissue Following Single Invasive and Repetitive Mild Injury

**Figure 4:**
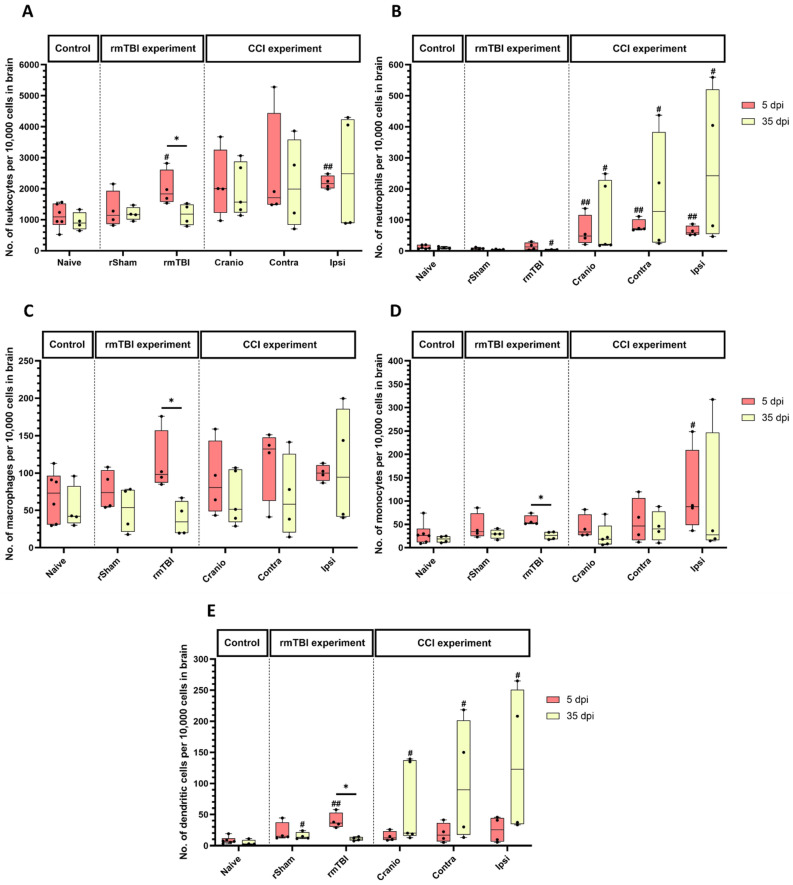
Analysis of innate immune cells in brain samples from rmTBI and CCI experiments. (**A**) Absolute count of peripheral immune cells in brain tissue per 10,000 gated cells, (**B**) absolute count of neutrophils per 10,000 CD45+ cells in brain tissue, (**C**) absolute count of peripheral macrophages per 10,000 CD45+ cells in brain tissue, (**D**) absolute count of monocytes per 10,000 CD45+ cells in brain tissue, and (**E**) absolute count of dendritic cells per 10,000 CD45+ cells in brain tissue. Results represent the median, minimum, and maximum. Data were analysed using the Friedman’s test (two-way analysis of variance by ranks), followed by the Dunn post hoc test; * *p* < 0.05, ^#^ *p* < 0.05, ^##^ *p* < 0.01. (*) represents a significant difference within an experimental group at different time points post injury, and (#) represents a significant difference between an experimental group and the naïve group at the same time point post injury. At 5 dpi: 6 animals for naïve, 4 animals for rSham, 4 animals for rmTBI, 4 animals for Cranio, and 4 animals for CCI. At 35 dpi: 4 animals for naïve, 4 animals for rSham, animals for rmTBI, 5 animals for Cranio, and 4 animals for CCI.

After rmTBI, at 5 dpi, there was an increase in the immune cell count (CD45+ cells) in the rmTBI group, compared to the corresponding naïve group (Figure 4A). There were differences in the rmTBI group across the two time points, with higher levels at 5 vs. 35 days. After rSham, no significant changes were observed after the procedure at 5 and 35 dpi. After CCI, there was a significant difference between the groups, at 5 dpi in the ipsilateral hemisphere of the CCI group, compared to the naïve group. The trend of an increase did not persist at 35 dpi. After craniotomy, no significant changes were observed at 5 and 35 dpi.

After rmTBI or the rSham procedure, there were few neutrophils detected in the brain tissue. The level was lower at 35 dpi after rmTBI (Figure 4B) when compared to the corresponding naïve group. In the CCI experiment, there were more neutrophils (Figure 4B) at 5 dpi in the Cranio group and the contralateral and ipsilateral hemispheres of the CCI group, when compared to the naïve group. This pattern continued at 35 dpi.

For peripheral macrophages (Figure 4C), the rmTBI data did not reveal any statistically significant differences between the experimental groups compared to the naïve at 5 dpi. However, there were differences in the rmTBI group across the two time points, with higher levels of macrophages at 5 vs. 35 days. In the CCI experiment, there were no significant differences between the groups.

The analysis of monocytes (Figure 4D) showed no significant differences in the rSham group and the rmTBI group when compared to the naïve group. However, there were differences in the rmTBI group across time points, with higher levels at 5 vs. 35 days. After CCI, there was an increase in the injured hemisphere vs. the naïve group at 5 days.

For dendritic cells (Figure 4E), the analysis of rmTBI groups showed a higher expression at 5 dpi in the rmTBI group vs. the naïve group. This trend did not persist at 35 dpi, but, at this time, there was a significant difference between the rSham group vs. the naïve group. The analysis showed differences in the rmTBI group across the two time points, with higher levels of dendritic cells at 5 vs. 35 days. In the CCI experiment, there was a significant difference at 35 dpi (Figure 4E), between the Cranio, Contra, and Ipsi groups, vs. the corresponding naïves.

**Figure 5:**
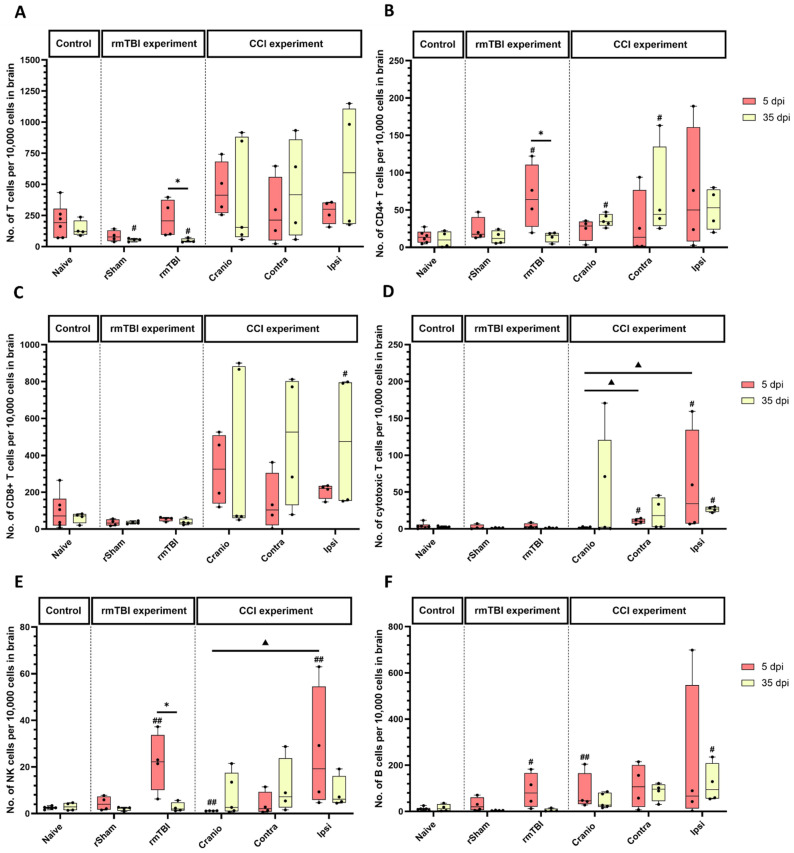
Analysis of adaptive immune cells in brain samples from rmTBI and CCI experiments. (**A**) Absolute count of T cells per 10,000 CD45+ cells in brain tissue, (**B**) absolute count of CD4+ T cells per 10,000 CD45+ cells in brain tissue, (**C**) absolute count of CD8+ T cells per 10,000 CD45+ cells in brain tissue, (**D**) absolute count of cytotoxic T cells per 10,000 CD45+ cells in brain tissue, (**E**) absolute count of NK cells per 10,000 CD45+ cells in brain tissue, and (**F**) absolute count of B cells per 10,000 CD45+ cells in brain tissue. Results represent the median, minimum, and maximum. Data were analysed using the Friedman’s test (two-way analysis of variance by ranks), followed by the Dunn post hoc test; * *p* < 0.05, ^#^ *p* < 0.05, ^##^ *p* < 0.01, ^▲^ *p* < 0.05. (*) represents a significant difference within an experimental group at different time points post injury, (#) represents a significant difference between an experimental group and the naïve group at the same time point post injury, and (▲) represents a significant difference between an experimental group and another experimental group at the same time point post injury. At 5 dpi: 6 animals for naïve, 4 animals for rSham, 4 animals for rmTBI, 4 animals for Cranio, and 4 animals for CCI. At 35 dpi: 4 animals for naïve, 4 animals for rSham, animals for rmTBI, 5 animals for Cranio, and 4 animals for CCI.

For cells associated with adaptive immunity, the analysis of the rmTBI experiments showed a decreased expression in the total T cell count in the rmTBI group and the rSham group at 35 dpi, when compared to the naïve group (Figure 5A). In addition, there were differences in the rmTBI group across the two time points, with more cells detected in the rmTBI group at 5 vs. 35 days. In the subsequent analysis of the T cell subpopulations, the CD4+ T cell count was significantly higher in the rmTBI group when compared to the naïve group, at 5 dpi (Figure 5B). Additionally, there were differences in the rmTBI group across the two time points, with higher levels at 5 days. In the CCI experiments, the CD4+ T cell count was higher in the Cranio and Contra groups at 35 days, but the increase did not reach significance in the ipsilateral hemisphere. For CD8+ T cells (Figure 5C), in CCI, there was an increase in CD8+ cells in the lesioned hemisphere at 35 days vs. the naïve group. Cytotoxic T cells (Figure 5D) showed a significant increase at 5 dpi in the contralateral hemisphere, and at 5 and 35 days in the lesioned hemisphere vs. the naïve group. Additionally, the analysis showed a significant difference between the Cranio group and the CCI group (both hemispheres) at 5 dpi.

The NK cell count was increased in the rmTBI group at 5 days (Figure 5E) when compared to the naïve group; the trend did not continue at 35 days. Additionally, there were differences in the rmTBI group across the two time points, with higher levels at 5 days. In the CCI experiment, there were statistically significant increases in the ipsilateral hemisphere of the CCI group when compared to the naïve group at 5 days. In addition, there was a significant decrease in the Cranio group vs. the naïve group at 5 dpi.

The B cell count (Figure 5F) showed a significant difference in the rmTBI group vs. the naïve group, at 5 dpi only. The analysis of the CCI experimental groups showed a significant increase in the B cell count at 5 dpi in the Cranio group when compared to the naïve group. At 35 dpi, there was a significant increase in the cell count in the lesioned hemisphere when compared to the naïve group.

**Figure 6:**
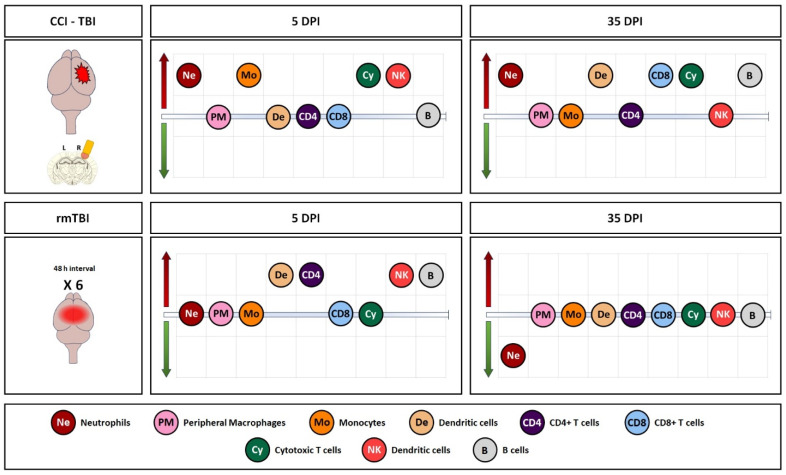
Summary of observed changes in the two models in brain samples. Red arrow indicates increase, and green arrow indicates decrease vs. naïves, and, for CCI, changes are represented only for the lesioned hemisphere.

An overview of all the observed changes in immune cells in the brain, expressed in percentages (in addition to the absolute counts presented in detail in the preceding graphs), in the two models is shown in Figure 6, Figure S1 (A—5 days and B—35 days), and Table S1, reflecting the global patterns of activation after the repeated mild injury and the more invasive single injury, and indicating a more persistent and intense response after CCI.

### 3.2. Immune System Response in Spleen, Blood, and Lymph Nodes Following Single Invasive and Repetitive Mild Injury

Tissues analysed included the spleen, blood, and lymph nodes (axillary, cervical, and inguinal). The latter were divided into upper and lower lymph nodes (ULN and LLN). Cells detected in the spleen and blood samples included neutrophils (CD45+, CD11b+, and Ly6G+), macrophages (CD45+ and F4/80), monocytes (CD45+, CD11b+, Ly6C+, and Ly6G-), dendritic cells (CD45+, CD11c+, and MHCII+), the total population of T cells (CD45+ and CD3+), CD4+ T cells (CD45+, CD3+, and CD4+), CD8+ T cells (CD45+, CD3+, and CD8a+), cytotoxic T cells (CD45+, CD3+, and CD335+), NK cells (CD45+, CD3-, and CD335+), and B cells (CD45+ and CD45R+). Cells identified in ULN and LLN samples included the total population of T cells, CD4+ T cells, CD8+ T cells, cytotoxic T cells, NK cells, and B cells.

**Figure 7:**
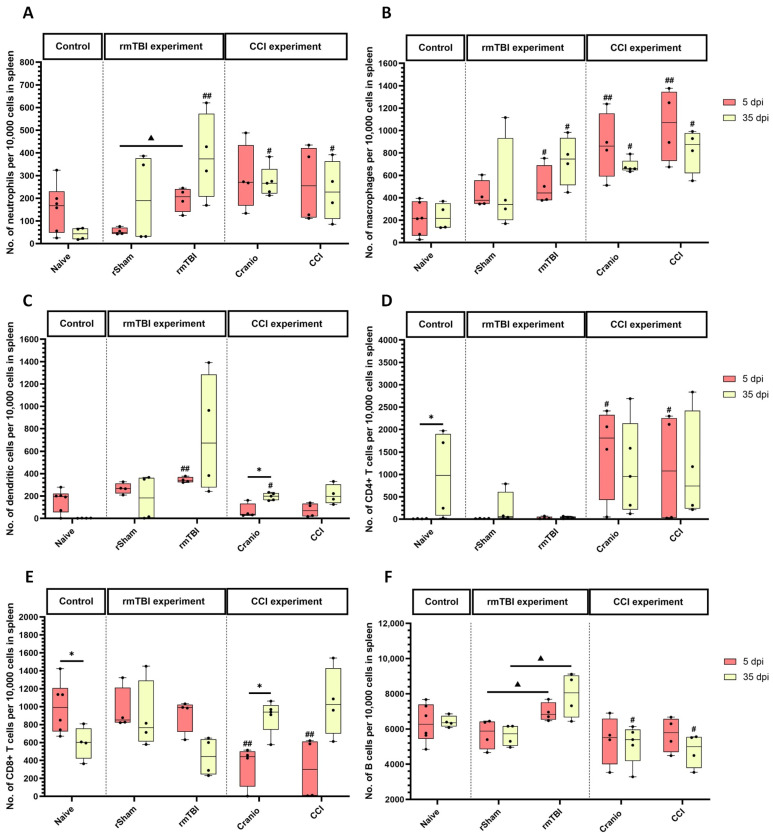
Spleen sample analysis from the rmTBI and CCI experiments. (**A**) Absolute count of neutrophils in the spleen per 10,000 gated cells, (**B**) absolute count of macrophages in the spleen per 10,000 gated cells, (**C**) absolute count of dendritic cells in the spleen per 10,000 gated cells, (**D**) absolute count of CD4+ T cells in the spleen per 10,000 gated cells, (**E**) absolute count of CD8+ T cells in the spleen per 10,000 gated cells, and (**F**) absolute count of B cells in the spleen per 10,000 gated cells. Results represent the median, minimum and maximum. Data were analysed using the Friedman’s test (two-way analysis of variance by ranks), followed by the Dunn post hoc test; * *p* < 0.05, ^#^ *p* < 0.05, ^##^ *p* < 0.01, ^▲^ *p* < 0.05. (*) represents a significant difference within an experimental group at different time points post injury, (#) represents a significant difference between an experimental group and the naïve group at the same time point post injury, and (▲) represents a significant difference between an experimental group and another experimental group at the same time point post injury. At 5 dpi: 4 animals for naïve, 4 animals for rSham, 4 animals for rmTBI, 4 animals for Cranio, and 4 animals for CCI. At 35 dpi: 6 animals for naïve, 4 animals for rSham, animals for rmTBI, 5 animals for Cranio, and 4 animals for CCI.

In the rmTBI experiment, the analysis of spleen tissue showed differences in neutrophils (Figure 7A) between the groups. At 35 dpi, the count was higher in the rmTBI group in comparison to the naïve group. Additionally, the neutrophil count was lower in the rSham group at 5 dpi when compared to the rmTBI group. In the CCI experiment, a significant increase in neutrophils (Figure 7A) was observed at 35 dpi between the CCI group vs. the naïve group, and the Cranio group vs. the naïve group.

In the rmTBI experiment, the macrophage population (Figure 7B) showed a significant increase at 5 dpi and also 35 dpi between the naïve group vs. the rmTBI group. In the CCI experiment, there was a significant increase in the macrophage population (Figure 7B) at 5 dpi in the CCI group when compared to the naïve group, as well as between the Cranio group when compared to the naïve group. The trend persisted at 35 dpi; the difference was significant between the CCI group vs. the naïve group, and the Cranio group vs. the naïve group.

For the monocyte count (Figure S2A), the analysis of the rmTBI experimental groups showed no significant differences at 5 dpi or 35 dpi between the naïve group vs. the rmTBI group, and between the naïve group vs. the rSham group. However, the monocyte counts were lower in the rmTBI group at 5 dpi when compared to the rSham group. In the CCI experiment, the analysis showed no significant differences at 5 dpi or 35 dpi between the experimental groups (Figure S2A).

As for dendritic cells (Figure 7C), the rmTBI group showed a higher expression at both time points; however, the difference was significant vs. the naïve group at 5 dpi. In the CCI experiment, there was no significant impact of the injury at both times post-injury. The analysis of the Cranio group showed a significant difference at 35 dpi when compared to the naïve group. In addition, there were differences in the Cranio group across the two time points, with higher levels of dendritic cells at 35 dpi.

For adaptive immunity, the total T cell count (Figure S2B) did not show a significant difference between the rmTBI experimental groups when compared to the naïve group. However, the cell count was significantly higher in the rSham group at 5 dpi when compared to the rmTBI group. The analysis of T cell subtypes showed a significant difference in CD4+ T cells (Figure 7D) in the naïve group across the two time points. In the CCI experiment, the analysis showed a significant increase in the CCI group and the Cranio group when compared to the naïve group at 5 dpi. The analysis of CD8+ T cells (Figure 7E) showed a significant difference in the naïve group across the two time points. In the CCI experiment, the CD8+ T cell count (Figure 7E) showed a significant decrease between the CCI and Cranio groups vs. the naïve group at 5 dpi. Additionally, there were differences in the Cranio group across the two time points, with a higher count at 35 dpi.

The analysis of cytotoxic T cells (Figure S2C) did not show differences between the rmTBI experimental groups. Similarly, the NK cell count (Figure S2D) showed no differences between the groups. In the CCI experiment, the cytotoxic T cell count (Figure S2C) was significantly lower in the Cranio group at 5 dpi vs. the naïve group. Additionally, there were differences in the Cranio group across the two time points, with a higher count at 35 dpi. The analysis of NK cells (Figure S2D) showed no distinct patterns or differences between groups.

B cells (Figure 7F) showed relatively higher cell counts when compared to other cell types. The analysis showed significant differences between the rmTBI group vs. the rSham group at 5 and 35 dpi. In the CCI experiment, there was a significant decrease in the B cell count (Figure 7F) at 35 dpi the CCI and the Cranio groups vs. the naïve group.

**Figure 8:**
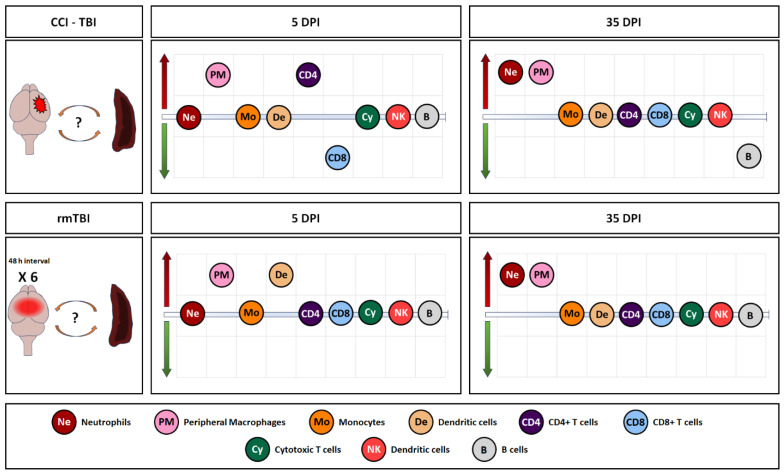
Summary of observed changes in the two models in spleen samples. Red arrow indicates increase, and green arrow indicates decrease vs. naïves.

An overview of all the observed changes in immune cells in the spleen, expressed in percentages (in addition to the absolute counts presented in detail in the preceding graphs), in the two models is shown in Figure 8, Figure S3 (A—5 days, and B—35 days), and Table S2, reflecting a complex immune response after the repeated mild injury and the more invasive single injury.

**Figure 9:**
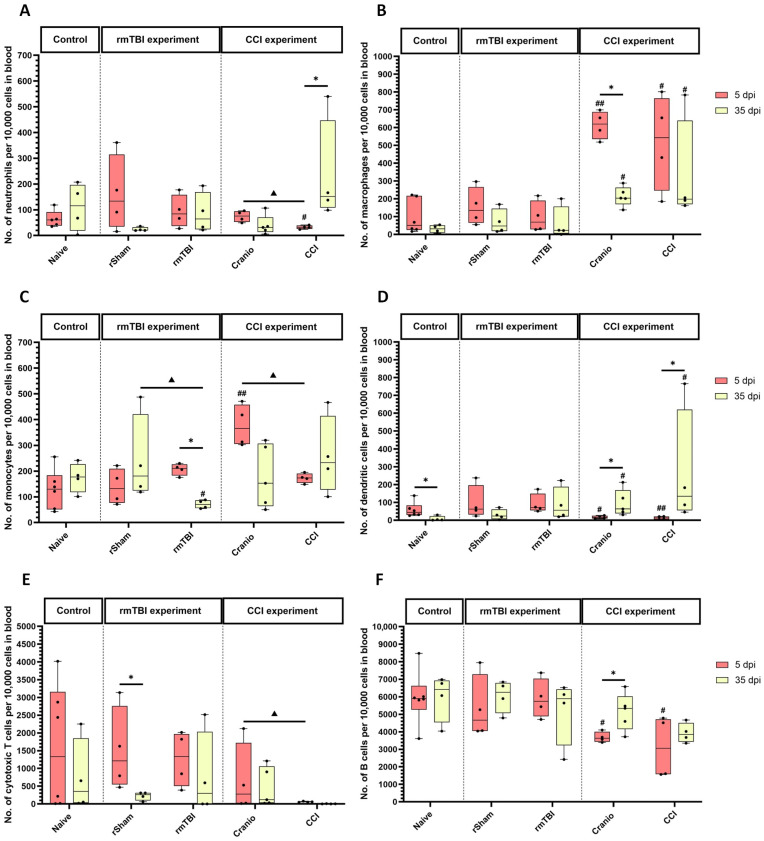
Blood sample analysis from the rmTBI and CCI experiments. (**A**) Absolute count of neutrophils in the blood per 10,000 gated cells, (**B**) absolute count of macrophages in the blood per 10,000 gated cells, (**C**) absolute count of monocytes in the blood per 10,000 gated cells, (**D**) absolute count of dendritic cells in the blood per 10,000 gated cells, (**E**) absolute count of cytotoxic T cells in the blood per 10,000 gated cells, and (**F**) absolute count of B cells in the blood per 10,000 gated cells. Results represent the median, minimum, and maximum. Data were analysed using the Friedman’s test (two-way analysis of variance by ranks), followed by the Dunn post hoc test; * *p* < 0.05, ^#^ *p* < 0.05, ^##^ *p* < 0.01, ^▲^ *p* < 0.05. (*) represents a significant difference within an experimental group at different time points post injury, (#) and (##) represents a significant difference between an experimental group and the naïve group at the same time point post injury, and (▲) represents a significant difference between an experimental group and another experimental group at the same time point post injury. At 5 dpi: 4 animals for naïve, 4 animals for rSham, 4 animals for rmTBI, 4 animals for Cranio, and 4 animals for CCI. At 35 dpi: 6 animals for naïve, 4 animals for rSham, animals for rmTBI, 5 animals for Cranio, and 4 animals for CCI.

In the analysis of blood samples, the neutrophil count (Figure 9A) did not show a significant difference between the rmTBI experimental groups when compared to the naïve group. In the CCI experiment, an analysis of neutrophils (Figure 9A) showed a significant decrease in the CCI group at 5 dpi when compared to the naïve group. In addition, the analysis showed a difference at 5 dpi, with lower levels in the CCI group vs. the Cranio group. At 35 dpi, there was an increase in the count of neutrophils in the CCI group when compared to levels at 5 dpi.

In the rmTBI experiment, there were no significant differences in macrophages (Figure 9B), dendritic cells (Figure 9D), total T cells (Figure S4A), CD4+ T cells (Figure S4B), CD8+ T cells (Figure S4C), NK cells (Figure S4D), and B cells (Figure 9F). For monocytes (Figure 9C), the analysis showed a significant decrease in the rmTBI group at 35 dpi vs. 5 dpi, and with the naïve group at 35 dpi. For cytotoxic T cells (Figure 9E), the analysis showed a difference in cell count in the Cranio group within the two time points, with higher levels at 5 dpi vs. 35 dpi, and a difference Cranio vs. CCI at 5 dpi.

In the CCI experiment, the analysis of macrophages (Figure 9B) showed a significant increase in cell count at 5 and 35 dpi in the CCI and Cranio groups vs. the naïve group. In addition, there was a significant difference in the macrophage count in the Cranio group within the two time points, with higher levels at 5 dpi vs. 35 dpi. As for monocytes (Figure 9C), the analysis showed a significant increase at 5 dpi in the Cranio group vs. the naïve group and the Cranio group vs. the CCI group. For dendritic cells (Figure 9D), the analysis showed a significant difference in cell count in the naïve, the Cranio, and the CCI groups within the two time points, with higher levels at 5 dpi vs. 35 dpi in the naïve group and higher levels at 35 dpi vs. 5 dpi in the Cranio and the CCI groups. In addition, the analysis showed a difference at 5 and 35 dpi between the Cranio and CCI groups vs. the naïve group. The analysis of T cells (Figure S4A) showed no significant differences between the CCI groups; however, the analysis of T cells subsets showed a significant increase in CD4+ T cell count (Figure S4B) at 5 dpi in the CCI group vs. the naïve group. For cytotoxic T cells, the analysis showed a significant difference at 5 dpi between the Cranio group vs. the CCI group. The analysis of NK cells showed an increase in the CCI group at 35 days vs. 5 dpi (Figure S4D). Lastly, the analysis of B cells showed significant decreases in the cell count in the CCI and Cranio groups at 5 dpi vs. the naïve group. Additionally, the analysis showed a significant difference in the cell count in the Cranio group within the two time points, with higher levels at 35 dpi vs. 5 dpi.

**Figure 10:**
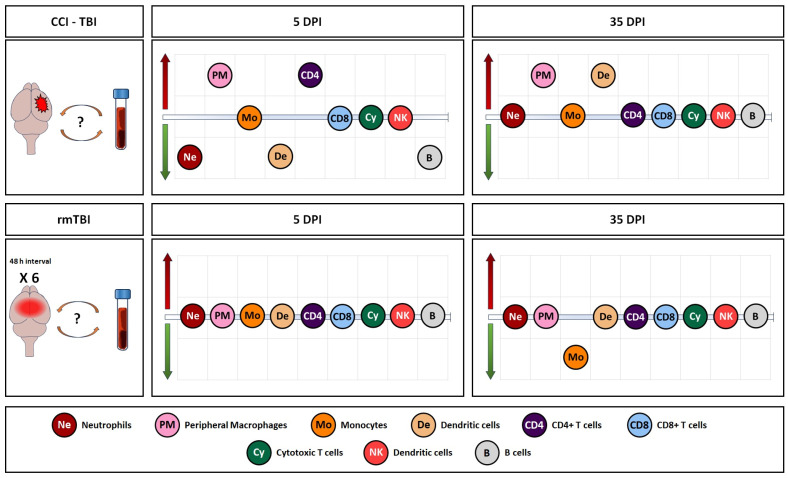
Summary of observed changes in the two models in blood samples. Red arrow indicates increase, and green arrow indicates decrease vs. naïves.

An overview of all the observed changes in immune cells in the blood, expressed in percentages (in addition to the absolute counts presented in detail in the preceding graphs), in the two models is shown in Figure 10, Figure S5 (A—5 days, B—35 days, and C—summary), and Table S3, reflecting the dynamic of the immune cell response after rmTBI and CCI.

The analysis of upper lymph nodes (ULN) in the rmTBI groups showed a significant decrease in the total T cell count (Figure S6A) in the rmTBI group at 35 dpi vs. 5 dpi, as well as the rmTBI group vs. the naïve group at 35 dpi. Additionally, the analysis showed a significant decrease in cell count at 5 and 35 dpi in the rmTBI group vs. the Cranio group. The analysis of CD4+ T cells (Figure S6B) showed no significant differences in the rmTBI experimental groups. CD8+ T cells (Figure S6C) showed a decrease in the rmTBI group at 35 dpi when compared to 5 dpi. In addition, the analysis showed a significant increase in cell count at 5 dpi in the CCI and the Cranio groups vs. the naïve group. For cytotoxic T cells (Figure S6D), the analysis showed a significant decrease in the rmTBI group at 35 dpi vs. 5 dpi. NK cells (Figure S6E) were increased in the rmTBI group at 35 dpi vs. 5 dpi. Additionally, there was a significant increase at the two time points in the rmTBI group vs. the Cranio group. A similar pattern was observed in the analysis of B cells (Figure S6F): a significant increase was observed in the rmTBI group at 35 dpi vs. 5 dpi. In addition, there was a significant increase in the rmTBI group vs. the Cranio group at 5 and 35 dpi. Furthermore, the analysis showed a significant increase in the B cell count at 35 dpi in the rmTBI group vs. the naïve group.

In CCI, the analysis showed no significant differences in the total T cell count (Figure S6A), CD4+ T cells (Figure S6B), CD8+ T cells (Figure S6C), cytotoxic T cells (Figure S6D), NK cells (Figure S6E), and B cells (Figure S6F).

For the lower lymph nodes (LLN), the analysis of the rmTBI group showed no significant differences in total T cells (Figure S8A), CD4+ T cells (Figure S8B), and B cells (Figure S8F). CD8+ T cells (Figure S8C) showed a difference between the rmTBI group vs. the naïve group at 5 dpi. For cytotoxic T cells (Figure S8D), there was a significant decrease in the naïve group at 35 dpi when compared to levels at 5 dpi. As for NK cells (Figure S8E), there was a significant increase in the rmTBI group at 35 dpi when compared to levels at 5 dpi. For CCI, the analysis of LLN showed no significant differences in total T cells (Figure S8A), CD4+ T cells (Figure S8B), CD8+ T cells (Figure S8C), cytotoxic T cells (Figure S8D), NK cells (Figure S8E), and B cells (Figure S8F).

An overview of all the observed changes in immune cells in the two models is shown in Figures S7 and S9, and Tables S4 and S5.

### 3.3. Cytokine Levels in Brain Tissue Following Single Invasive or Repetitive Mild Injury

A multiplex cytokine array was used to detect 10 cytokines/chemokines in brain samples collected from the various experimental groups. The levels of several cytokines, including IFN-γ, IL-1β, IL-2, IL-10, IL-12p70, and TNF-α, were below the lower limit of quantification (LLOQ). However, the levels of IL-4, IL-5, IL-6, and KC/GRO (CXCL1) were above the LLOQ, allowing for their quantification.

**Figure 11:**
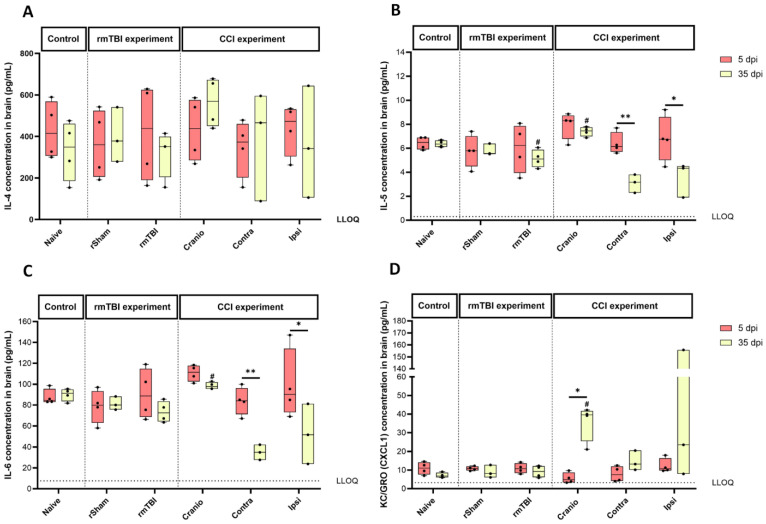
Different cytokine levels (pg/mL) in brain samples at 5 and 35 dpi. (**A**) Interleukin-4 (IL-4), (**B**) Interleukin-5 (IL-5), (**C**) Interleukin-6 (IL-6), and (**D**) keratinocyte chemoattractant (KC)/human growth-regulated oncogene (GRO) (chemokine ligand 1) (KC/GRO CXCL1). Results represent the median, minimum, and maximum. Data were analysed using the Friedman’s test (two-way analysis of variance by ranks), followed by the Dunn post hoc test, and ^#^ *p* < 0.5, * *p* < 0.05, ** *p* < 0.01. (*) represents a significant difference within an experimental group at different time points post injury, and (#) represents a significant difference between an experimental group and the naïve group at the same time point post injury. At 5 dpi: 4 animals for naïve, 4 animals for rSham, 4 animals for rmTBI, 4 animals for Cranio, and 4 animals for CCI. At 35 dpi: 4 animals for naïve, 4 animals for rSham, animals for rmTBI, 4 animals for Cranio, and 4 animals for CCI. LLOQ: lower limit of quantification.

In rmTBI, there were no significant differences between the groups in the levels of IL-4 (Figure 11A), IL-6 (Figure 11C), and KC/GRO (CXCL1) (Figure 11D); therefore, there was no specific response to injury at the two time points. The analysis of IL-5 (Figure 11B) showed a decrease at 35 dpi between the rmTBI group vs. the naïve group. In the CCI experiment, IL-4 levels (Figure 11A) did not differ between the groups. The analysis of IL-5 (Figure 11B) showed significant differences between the groups; differences between the Cranio vs. the naïve groups at 35 dpi, and within the ipsilateral and contralateral hemispheres when considering the time after the injury. For IL-6, there were differences in between the Cranio group vs. the naïve group at 35 dpi, and within the ipsilateral and contralateral hemispheres when considering time (Figure 11C). As for KC/GRO (CXCL1) (Figure 11D), differences were observed between the Cranio group vs. the naïve group at 35 dpi and within the Cranio group when considering time after the procedure.

## 4. Discussion

The persistent activation of an inflammatory response plays a central role in the TBI outcome. The inflammatory reaction to TBI and the initiation of a general immune activation is triggered acutely by the release of different cellular components from damaged and dying cells (e.g., ATP and high mobility group box 1 protein [HGMB1]), described as damage-associated molecular patterns (DAMPs) [36,37], which can activate Toll-like receptors (TLR) on immune cells and lead to the secretion of cytokines and chemokines; this further activates resident glial cells and increases the migration of peripheral immune cells to the injury site. A prolonged inflammatory response can be detected in brain parenchyma in humans and rodent experimental models after a single TBI [38,39,40] and also repetitive TBI [41]. Raised levels of cytokines can also be detected in the patients’ plasma, even at 12 months after a mild TBI [42].

For the peripheral cellular immune response, neutrophils are the first cells that show changes in blood samples and infiltrate the brain, with an increased absolute cell count in the first 24 h following injury, which continues for a few days [43,44]. The analysis of the rmTBI tissue showed the increased infiltration of leukocytes in brain tissue, with dendritic cells, CD4+ T cells, NK cells, and B cells that were significantly increased at the acute phase after injury. These observations do not align with what is reported in studies in juvenile animals [32] and adult animals [20,45], as neutrophils are the type of cells usually detected as early as a few minutes after injury, and they peak 2 h after injury in the subarachnoid and subdural spaces. The infiltration reaches maximum levels in the brain 24 to 48 h after cortical injury [46], then decreases gradually until it reaches very low levels at 7 days [20] in adult brain, and 2 weeks in juvenile brain [32]. The analysis of brain tissue in the CCI experiments showed an increased infiltration of leukocytes across all groups (craniotomy and the two hemispheres), including neutrophils in the acute phase; therefore, this was not a response confined just to the injured side. The unexpectedly higher neutrophil count observed in the subacute phase was accompanied by changes in the peripheral organs studied, notably an increased neutrophil count in the spleen. The origin and role of these neutrophils during the subacute phase remain unclear.

Neutrophil infiltration is followed by the increase in monocytes/peripheral macrophages in adult animals [20,45]. The data from the rmTBI study show no significant increase in the monocyte count in the acute phase after injury. The data from the CCI study show an increase in the ipsilateral hemisphere of the injured mice in the acute phase; however, the available data cannot elucidate if this increase affects specific subsets of monocytes [47].

As for dendritic cells, there are fewer studies in the context of TBI compared to other immune cells. Evidence suggests their activation and redistribution in the peripheral immune organs [48] and their increased presence in the brain post-injury [49]. Our data in the rmTBI model suggest an increase in dendritic cell count in the acute phase following injury. This increase was also observed in the spleen, indicating a potential shift or redistribution of dendritic cells. After CCI, an increase in dendritic cell count was observed in both the ipsilateral and contralateral hemispheres, as well as in the craniotomy group, during the subacute phase after injury. This increase was also observed in blood samples, implying a shift towards the increased production of dendritic cells in the bone marrow.

The analysis of NK cells in the rmTBI tissues showed a significant increase in the acute phase, which returned to normal levels in the subacute phase. As for the CCI tissue, a significant change was observed in the acute phase, specifically, in the injured ipsilateral side. Interestingly, the craniotomy group showed a significant decrease in cell count during the acute phase after the procedure. Clinical studies have reported a reduction in absolute numbers of NK cells in blood samples of patients at days/weeks following mild, moderate, and severe injuries [47]. Some hypothesise it to be a protective mechanism to prevent autoimmunity and minimise neuroinflammation [50]. In vitro studies have demonstrated the effect of NK cells in killing homeostatic microglia rather than activated microglia [51]. In addition, resident NK cells affect the induction of Th17 function (mediated by microglia) [52]. Another explanation for the NK cell decline is apoptosis due to high levels of serum glucocorticoids which may have occurred after injury [53,54].

As for adaptive immune cells, the rmTBI tissue showed a significant decrease in total T cell count in the subacute phase, an increased CD4+ T cell count during the acute phase, and no changes in the CD8+ T cell and cytotoxic T cell counts. Changes in adaptive immune cells have been observed in other TBI models [24,55,56]. Studies have reported a reduced percentage and absolute number of circulating T lymphocytes between 1–4 days after injury. This reduction involved both CD4+ T cells and CD8+ cytotoxic T cells [47,57]. Adaptive immune cells peak around 5–11 days after injury [24,55,56]. Their role is not fully defined but evidence suggests the harmful effects of cytotoxic T cells. A population of CD8+ T cells which expressed GrB, a protease with pro-apoptotic activity and a marker of cytotoxic immune cells, infiltrated the brain 24 h after open skull weight drop injury, and caused neuronal apoptosis through the activation of the caspase-3/poly ADP ribose polymerase (PARP) pathways [58].

Interestingly, our analysis implied the presence of the CD3^+^, CD4^−^, and CD8^−^ subtype of T cells in all groups, (not identified by the designed panel). Studies refer to it as double-negative (DN) T cells or gamma delta T cells. This type of cell was reported previously in TBI, in a study that investigated in adult male C57/BL6 mice the response of immune cells up to 8 months after CCI; the presence of these cells was substantial at 32 weeks after injury [55]. The exact role of this cell population in TBI is not known.

Our analysis of rmTBI and CCI tissues showed significant changes in the B cell count during the acute and subacute phases—specifically, in the rmTBI group in the acute phase, in the craniotomy group in the acute phase, and in the ipsilateral hemisphere of the CCI group in the subacute phase. The involvement of B cells after brain injury has not been as well-characterised as that of other immune cell types. The data show the presence of autoantibodies against CNS proteins after TBI [59]. Some argue that the presence of such antibodies has a regulatory effect after injury [55,60], whereas others suggest a negative impact [61,62,63].

The analysis of the peripheral organs of rmTBI animals indicated an increase in neutrophils, macrophages, and dendritic cell counts in the spleen, an increase in CD8+ T cells and B cells in the cervical and axillary lymph nodes, and an increase in CD8+ T cells in the inguinal lymph nodes. This implies the involvement of lymphatic organs in mobilising immune cells following injury, similar to what was reported in other studies that examined brain injury in adult animals [21,64,65]. In a focal cerebral ischemia study in rats and mice, the results showed the activation of macrophages 24 h after the procedure. A microarray analysis showed the involvement of VEGF-C/VEGFR3 signalling; the blocking of this pathway reduced the presence of pro-inflammatory macrophages in cervical lymph nodes, and, subsequently, reduced infarction after the procedure.

In the CCI model, the analysis of the spleen showed an increased macrophage count in the craniotomy and injured groups, and a decrease in CD8+ T cells, in the acute phase. In addition, the blood sample analysis showed a significant decrease at the acute point in the count of neutrophils, dendritic cells, NK cells, and B cells, and an increase in the macrophage and dendritic cell count in the subacute phase. The changes in leukocyte populations in the spleen and blood did partially correlate with changes in the brain.

The major pro-inflammatory cytokines released acutely post-injury are interleukin-1β (IL-1β), interleukin-6 (IL-6), and tumour necrosis factor alpha (TNFα), while the anti-inflammatory cytokines include interleukin-10 (IL-10) and transforming growth factor beta (TGFβ). Frugier et al. (2010) [66] reported a very rapid rise (both mRNA and protein levels) in IL-1β, IL-6, IL-8, and TNF-α in post-mortem human brains acutely after injury. In a study in paediatric TBI, Ryan et al. (2022) [67] showed complex changes in the blood cytokine profile, in mild TBI and severe TBI; irrespective of severity, in the first 4 days after injury, all children had increased IL-6—with higher levels seen in severe TBI. The mTBI group had significantly increased IFN-γ versus controls, but IFN-γ levels were decreased in severe injury compared to controls. IL-8, IL-10, IL-17A, and TNF-α were significantly decreased in mild TBI compared to controls.

In the rmTBI study in our juvenile mice, IL-5 levels, a pro-inflammatory cytokine with modulatory roles on eosinophils, and also B cells (antibody secretion and class switching) were lower in the injured tissue vs. naïve tissue. Interestingly, in adult mice, rmTBI studies by Algamal et al. (2019) [68] have shown in the injured brain parenchyma trends towards decreases in IL-6, IL-2, IFN-γ, and IL-1β. In adult rats with rmTBI, a study reported higher brain tissue levels of IL-6, TNF-α, and IL-10 at 1–2 weeks after injury [69]. Corrigan et al. (2017) [70] also reported, in rmTBI in adult rats, trends towards increases in IL-6, TNF-α, IFN-γ, and IL-17a, at 1 week. Other studies reported an increase in IL-5 and IL-6 in addition to other cytokines and chemokines including CCL2, CCL3, CCL4, CCL9, IL-1β, TGFβ, IL-10, IL-18, IFN-γ, TNF-α, IP-10, and MIP-1a levels in brain tissue [71,72,73,74,75,76]. Thus, the trend we see in the juvenile brain towards decreased levels of IL-5 and IL-6 is in contrast with what is reported in the literature in the adult brain. In studies in TBI patients, increased levels of TNF-α and IL-10 in serum/plasma and cerebrospinal fluid (CSF) samples [77,78,79], and IL-6 and IL-8 in CSF samples [80,81,82] have been reported.

To summarise our results, repetitive mild injury in the immature brain caused an infiltration in leukocytes from the periphery, including dendritic cells, T cells subsets, NK cells, and B cells. An analysis of the peripheral organs showed an increase in macrophages, neutrophils, dendritic cells, and B cell count in the spleen, and an increase in CD8+ T cells and B cells in the cervical and axial lymph nodes. There were no changes in leukocyte populations in the blood. Invasive injury in the immature brain also caused the brain infiltration of immune cells including neutrophils, monocytes, dendritic cells, CD4+ T cells, CD8+ T cells, cytotoxic T cells, NK cells, and B cells. Changes in leukocyte populations in the peripheral immune organs were observed, such as an increase in macrophages and neutrophils in the spleen, a decrease in NK cells and B cells, and increase in dendritic cells in blood. Overall, there was, therefore, a clear activation of an immune response, and changes were detected in the immature brain with variable intensity depending on the type of injury.

An important observation in both injury models in these juvenile animals is that the control procedures (repeated anaesthesia for rmTBI or craniotomy for CCI) induced a response of the immune system, for some of the cells analysed, when compared to naïve animals. We had noticed the significant impact of these procedures in the juvenile brain in our previous study, focused on the microglial and astrocytic response of animals [33], and, using a transcriptomic analysis of brain tissue, we showed that these control procedures induce significant changes related to cellular senescence and inflammation, which are quite different from the impact of the brain injury per se. Inhalation anaesthetics, of which isoflurane is an example, have been shown to suppress innate immunity by impairing or suppressing neutrophil adhesion, monocytes, macrophages, and the cytostatic activity of NK cells, and they can also modulate adaptive immunity, such as reducing the proliferation of CD4+ and CD8+ T cells and inducing the apoptosis of T and B lymphocytes [83,84]. There is evidence that isoflurane could also decrease the systemic cytokine response, e.g., the levels of IL-1β, TNF-α, IL-6, IL-8, and IL-10. These observations suggest a general non-specific injury or stress response component which is significant in these juvenile animals, irrespective of the direct impact of a force on the brain tissue.

To conclude, our findings demonstrate the clear activation of various elements of a peripheral immune response in two types of injury of the immature brain. The observations indicate that the immune response depends on the injury type and has some similarities, but also differences with what has been reported in adult animals.

## Figures and Tables

**Table 1:** FACS panel for innate immunity. It explored a variety of cells including total leukocytes infiltrating the brain, peripheral macrophages, monocytes, neutrophils, and dendritic cells.

Antigen	Fluorochrome	Laser	Wavelength	Manufacturer
CD45	FITC	B	530/30	Miltenyi Biotec (Bergisch Gladbach, Germany)
F4/80	APC	R	670/14	Biolegend
Live/Dead	Zombie NIR	R	780/60	Biolegend
Ly6C	VioBlue	V	450/50	Miltenyi Biotec
Ly6G	BV605	V	610/20	Biolegend
MHC II	BV711	V	710/50	Biolegend
CD11c	PE	YG	582/10	Miltenyi Biotec
CD11b	PE-Vio615	YG	610/20	Miltenyi Biotec

**Table 2:** FACS panel for adaptive immunity. It explored a variety of cells including B cells, total T cells, CD4+ T cells, CD8+ T cells, cytotoxic T cells, and natural killer (NK) cells.

Antigen	Fluorochrome	Laser	Wavelength	Manufacturer
CD45	FITC	B	530/30	Miltenyi Biotec
CD8a	PerCP-Vio700	B	695/40	Miltenyi Biotec
CD3	APC	R	670/14	Biolegend
Live/Dead	Zombie NIR	R	780/60	Biolegend
CD45R/B219	VioGreen	V	525/49	Miltenyi Biotec
CD19	BV785	V	780/60	Biolegend
CD335/NKp46	PE	YG	582/10	Miltenyi Biotec
CD4	PE-Vio615	YG	615/20	Miltenyi Biotec

## Data Availability

The data are available upon request from the authors.

## Supplementary Materials

The following supporting information can be downloaded at: https://www.mdpi.com/article/10.3390/cells13191612/s1, Figure S1: Percentages of immune cells in brain tissue; Table S1: Summary of all the observed changes in immune cells in the two models in brain samples when compared to the naïve group; Figure S2: Spleen sample analysis from the rmTBI and CCI experiments; Figure S3: Percentages of immune cells in spleen tissue; Table S2: Summary of all the observed changes in immune cells in the two models in spleen samples when compared to the naïve group; Figure S4: Blood sample analysis from the rmTBI and CCI experiments; Figure S5: Percentages of immune cells in blood tissue; Table S3: Summary of all the observed changes in immune cells in the two models in blood samples when compared to the naïve group; Figure S6: Axillary and cervical lymph nodes sample analysis from the rmTBI and CCI experiments; Figure S7: Upper lymph nodes analysis from the rmTBI and CCI experiments; Table S4: Summary of all the observed changes in immune cells in the two models in upper lymph nodes samples when compared to the naïve group; Figure S8: Inguinal lymph nodes sample analysis from the rmTBI and CCI experiments; Figure S9: Lower lymph nodes analysis from the rmTBI and CCI experiments; Table S5: Summary of all the observed changes in immune cells in the two models in lower lymph nodes samples when compared to the naïve group.

## Abbreviations

ULN	upper lymph nodes
LLN	lower lymph nodes
IL	interleukin
CNS	central nervous system
ROS	reactive oxygen species
