# Supplementary material for: The Immune Response in Two Models of Traumatic Injury of the Immature Brain
Source: Cells. 2024 Sep 26;13(19):1612. doi: 10.3390/cells13191612 (PMC11475908; doi:10.3390/cells13191612)
Supplement: Supplementary file 1 [file cells-13-01612-s001.zip › cells-3163313-supplementary.pdf]

Immune system response in brain tissue

Figure S1

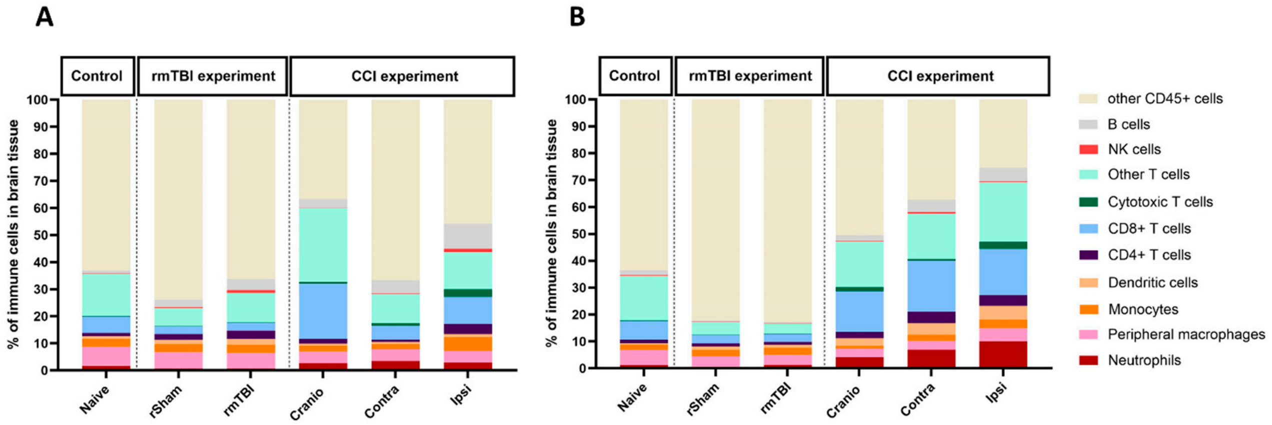

Figure S1: Brain sample analysis from the rmTBI and CCI experiments. Percentages of immune cells in brain tissue (A) at 5 dpi and (B) at 35 dpi.

Table S1

Table S1: Summary of all the observed changes in immune cells in the two models in brain samples when compared to the naïve group. (↑S): significant increase, (↓S): significant decrease, (-): non-significant difference, (I): Ipsi, (C): Contra.

Cell type	rSham		rmTBI		Cranio		CCI	
	Acute phase	Subacute phase	Acute phase	Subacute phase	Acute phase	Subacute phase	Acute phase	Subacute phase
Immune cells	-	-	↑S	-	-	-	↑S (I)	-
Neutrophils	-	-	-	↓S	↑S	↑S	↑S (C, I)	↑S (C, I)
Macrophages	-	-	-	-	-	-	-	-
Monocytes	-	-	-	-	-	-	↑S (I)	-
Dendritic cells	-	↑S	↑S	-	-	↑S	-	↑S (C, I)
Total T cells	-	↓S	-	↓S	-	-	-	-
CD4+ T cells	-	-	↑S	-	-	↑S	-	↑S (C)
CD8+ T cells	-	-	-	-	-	-	-	↑S (I)
Cytotoxic T cells	-	-	-	-	-	-	↑S (C, I), s*	↑S (I)
NK cells	-	-	↑S	-	↓S	-	↑S (I), s*	-
B cells	-	-	↑S	-	↑S	-	-	↑S (I)

*** Represent a significant difference between an injured group and its sham group at the same time point post injury.**

Immune system response in spleen

Figure S2

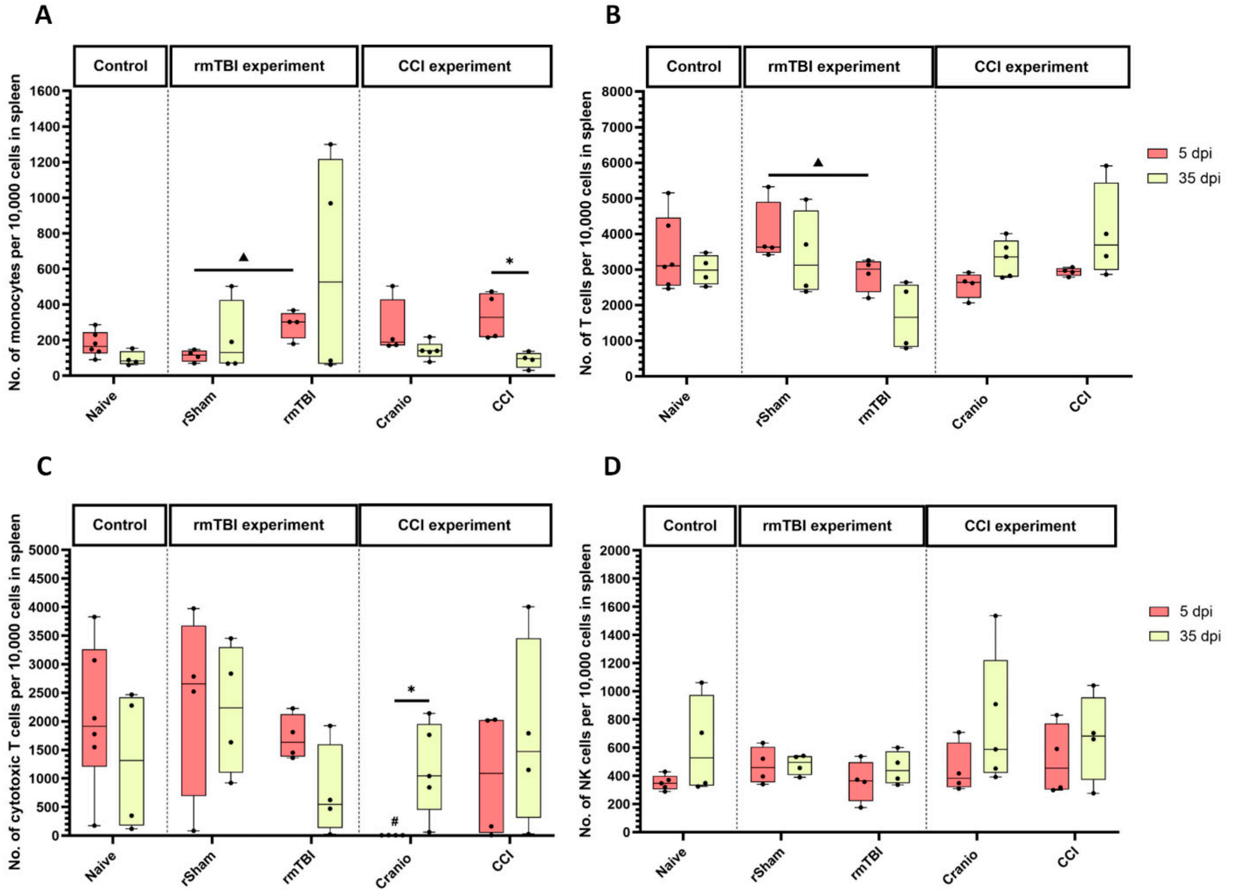

Figure S2: Spleen sample analysis from the rmTBI and CCI experiments. (A) Absolute count of monocytes in the spleen per 10,000 gated cells, (B) Absolute count of T cells in the spleen per 10,000 gated cells, (C) Absolute count of cytotoxic T cells in the spleen per 10,000 gated cells, (D) Absolute count of NK cells in the spleen per 10,000 gated cells. Results represent the median, minimum and maximum. Data were analysed using the Friedman's test (two-way analysis of variance by ranks) followed by the Dunn post-hoc test; *P<0.05, #P<0.05, ▲P<0.05. (*) represent a significant difference within an experimental group at different time points post injury, (#) represent a significant difference between an experimental group and the naïve group at the same time point post injury, (▲) represent a significant difference between an experimental group and another experimental group at the same time point post injury. At 5 dpi: 4 animals for naive, 4 animals for rSham, 4 animals for rmTBI, 4 animals for cranio and 4 animals for CCI. At 35 dpi: 4 animals for naive, 4 animals for rSham, animals for rmTBI, 5 animals for cranio and 4 animals for CCI.

Figure S3

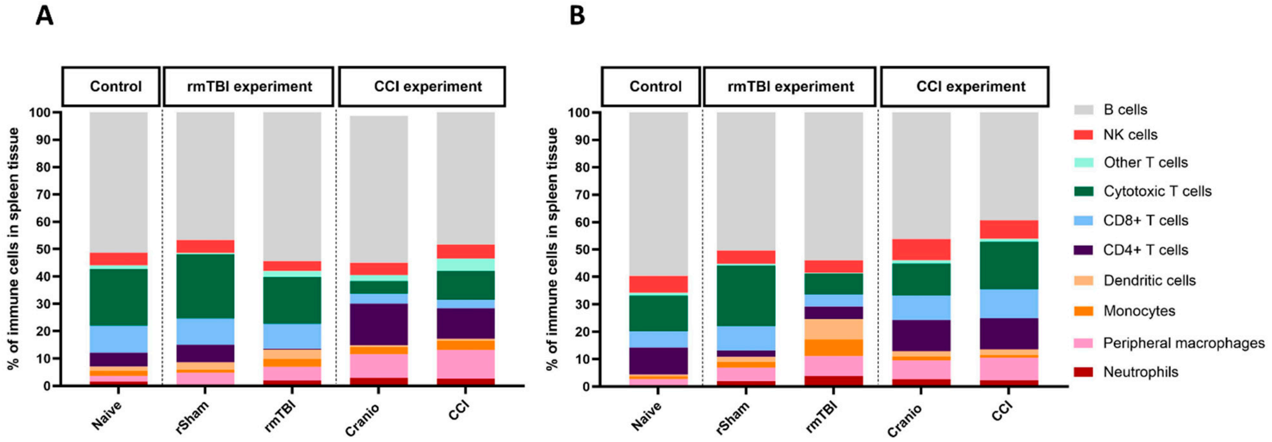

Figure S3: Spleen sample analysis from the rmTBI and CCI experiments. Percentages of immune cells in spleen tissue (A) at 5 dpi and (B) at 35 dpi.

Table S2

Table S2: Summary of all the observed changes in immune cells in the two models in spleen samples when compared to the naïve group. (↑S): significant increase, (↓S): significant decrease, (-): non-significant difference.

Cell type	rSham		rmTBI		Cranio		CCI	
	Acute phase	Subacute phase	Acute phase	Subacute phase	Acute phase	Subacute phase	Acute phase	Subacute phase
**Neutrophils**	-	-	s*	↑S	-	↑S	-	↑S
**Macrophages**	-	-	↑S	↑S	↑S	↑S	↑S	↑S
**Monocytes**	-	-	s*	-	-	-	-	-
**Dendritic cells**	-	-	↑S	-	-	↑S	-	-
**Total T cells**	-	-	-	s*	-	-	-	-
**CD4+ T cells**	-	-	-	-	↑S	-	↑S	-
**CD8+ T cells**	-	-	-	-	↓S	-	↓S	-
**Cytotoxic T cells**	-	-	-	-	↓S	-	-	-
**NK cells**	-	-	-	-	-	-	-	-
**B cells**	-	-	s*	s*	-	↓S	-	↓S

*** Represent a significant difference between an injured group and its sham group at the same time point post injury.**

Immune system response in blood

Figure S4

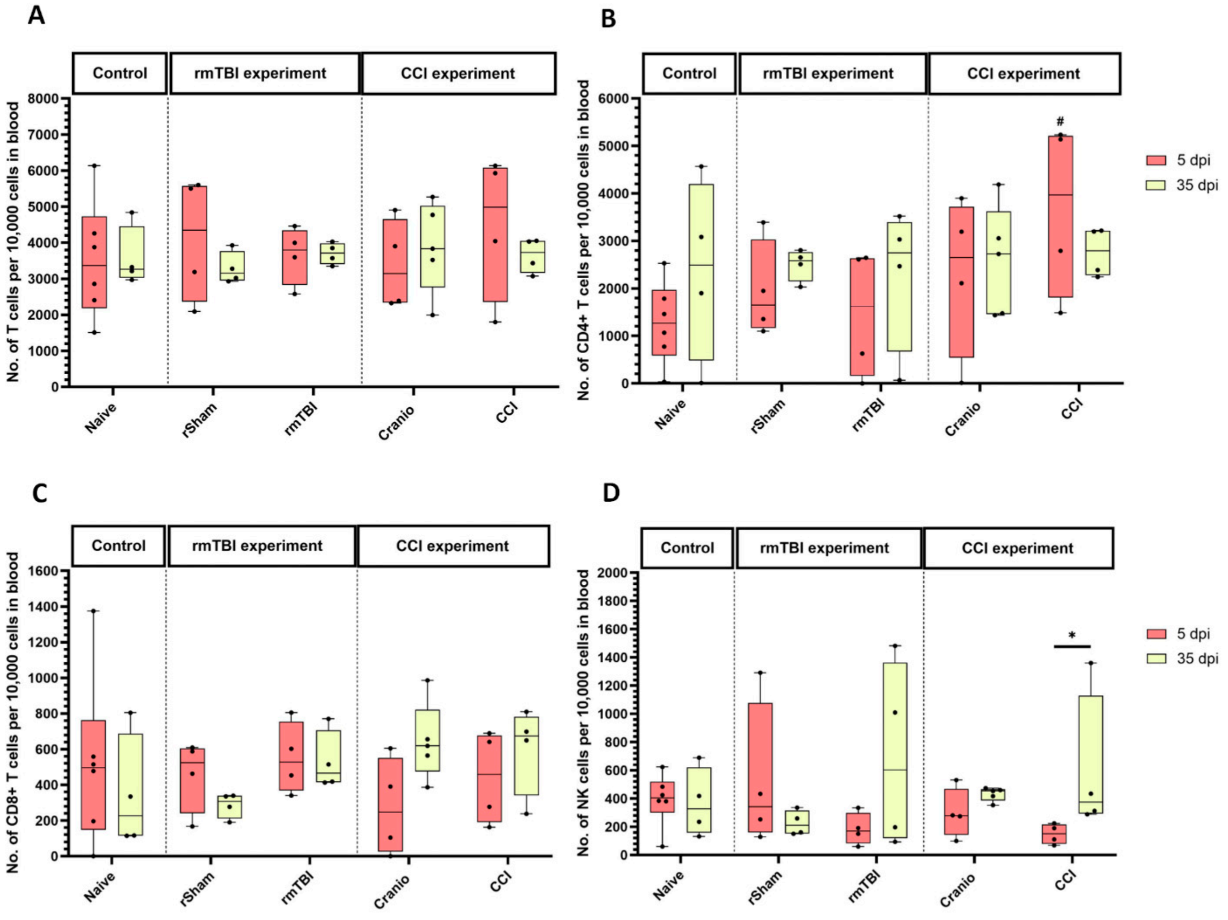

Figure S4: Blood sample analysis from the rmTBI and CCI experiments. (A) Absolute count of T cells in the blood per 10,000 gated cells, (B) Absolute count of CD4+ helper T cells in the blood per 10,000 gated cells, (C) Absolute count of CD8+ T cells in the blood per 10,000 gated cells, (D) Absolute count of NK cells in the blood per 10,000 gated cells. Results represent the median, minimum and maximum. Data were analysed using the Friedman's test (two-way analysis of variance by ranks) followed by the Dunn post-hoc test; *P<0.05, #P<0.05. (*) represent a significant difference within an experimental group at different time points post injury, (#) represent a significant difference between an experimental group and the naïve group at the same time point post injury. At 5 dpi: 4 animals for naive, 4 animals for rSham, 4 animals for rmTBI, 4 animals for cranio and 4 animals for CCI. At 35 dpi: 4 animals for naive, 4 animals for rSham, animals for rmTBI, 5 animals for cranio and 4 animals for CCI.

Figure S5

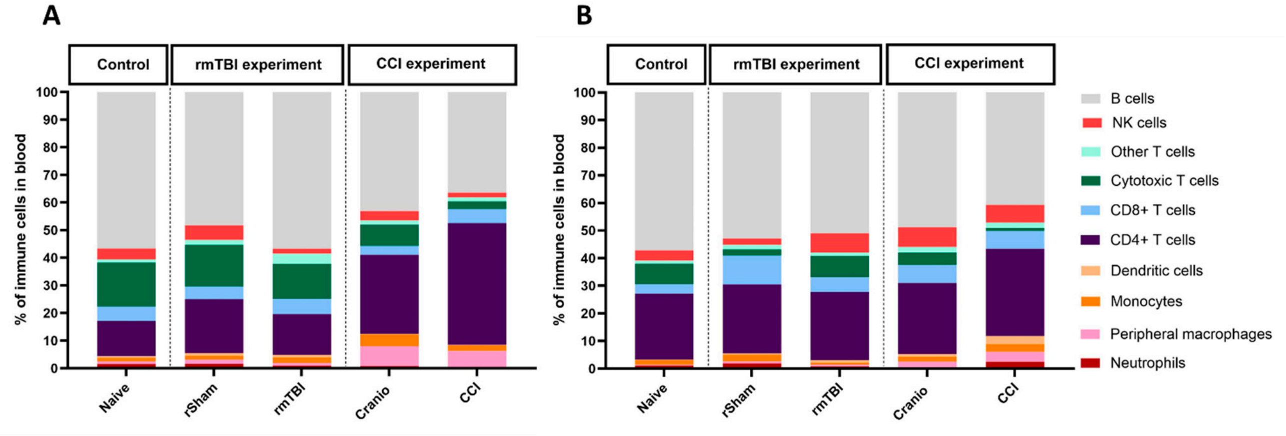

Figure S5: Blood sample analysis from the rmTBI and CCI experiments. Percentages of immune cells in blood tissue (A) at 5 dpi and (B) at 35 dpi.

Table S3

Table S3: Summary of all the observed changes in immune cells in the two models in blood samples when compared to the naïve group. (↑S): significant increase, (↓S): significant decrease, (-): non-significant difference.

Cell type	rSham		rmTBI		Cranio		CCI	
	Acute phase	Subacute phase	Acute phase	Subacute phase	Acute phase	Subacute phase	Acute phase	Subacute phase
Neutrophils	-	-	-	-	-	-	↓S, s*	-
Macrophages	-	-	-	-	↑S	↑S	↑S	↑S
Monocytes	-	-	-	↓S, s*	↑S	-	s*	-
Dendritic cells	-	-	-	-	↓S	-	↓S	↑S
Total T cells	-	-	-	-	-	-	-	-
CD4+ T cells	-	-	-	-	-	-	↑S	-
CD8+ T cells	-	-	-	-	-	-	-	-
Cytotoxic T cells	-	-	-	-	-	-	s*	-
NK cells	-	-	-	-	-	-	-	-
B cells	-	-	-	-	↓S	-	↓S	-

*** Represent a significant difference between an injured group and its sham group at the same time point post injury.**

Immune system response in upper lymph node (ULN)

Figure S6

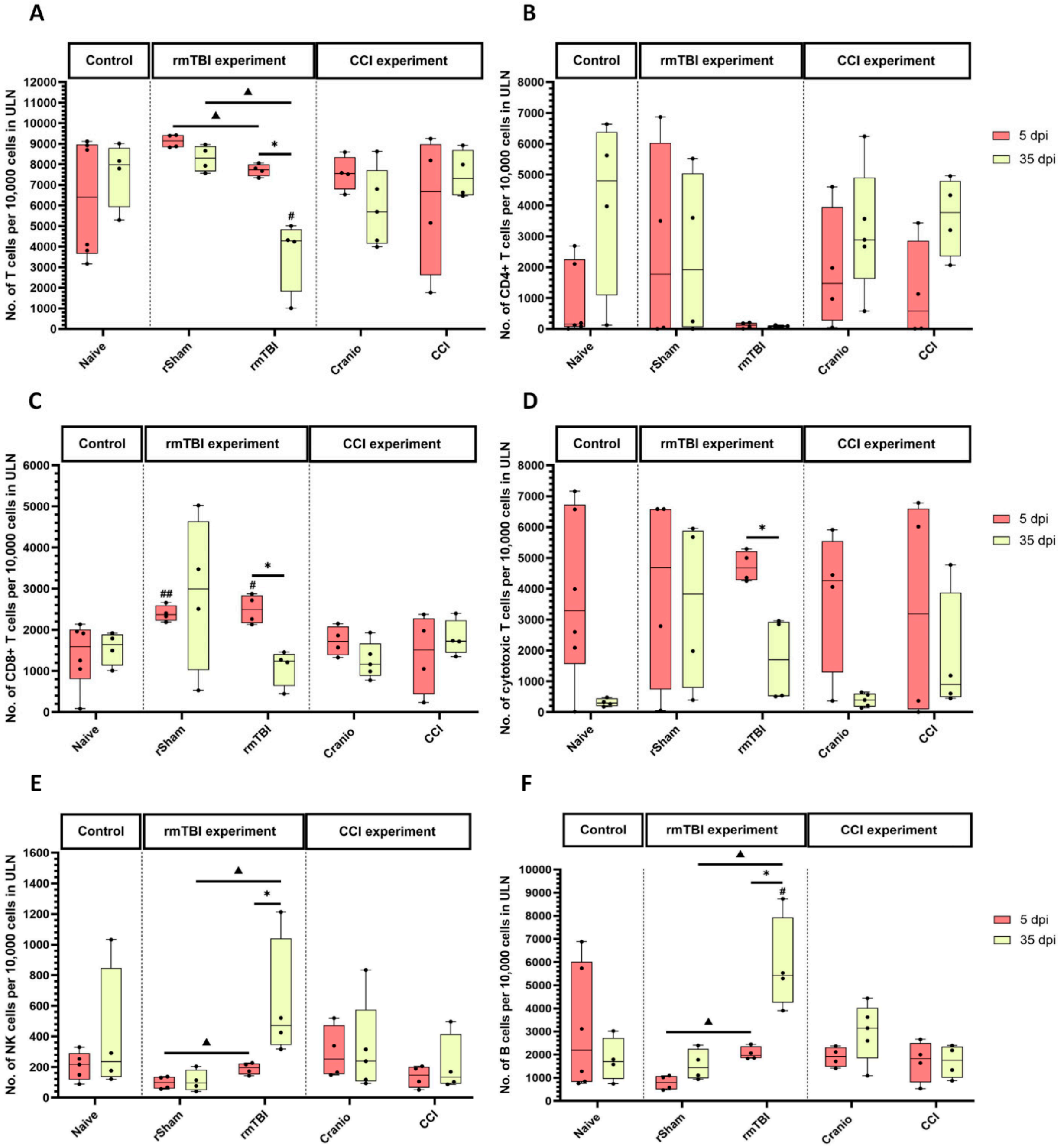

Figure S6: Axillary and cervical lymph nodes sample analysis from the rmTBI and CCI experiments. (A) Absolute count of T cells in the upper lymph nodes (ULN) per 10,000 gated cells, (B) Absolute count of CD4+ helper T cells in the ULN per 10,000 gated cells, (C) Absolute count of CD8+ T cells in the ULN per 10,000 gated cells, (D) Absolute count of cytotoxic T cells in the ULN per 10,000 gated cells, (E) Absolute count of NK cells in the ULN per 10,000 gated cells, (F) Absolute count of B cells in the ULN per 10,000 gated cells. Results represent the median, minimum and maximum. Data were analysed using the Friedman's test (two-way analysis of variance by ranks) followed by the Dunn post-hoc test; *P<0.05, #P<0.05, ##P<0.01, ▲P<0.05. (*) represent a significant difference within an experimental group at different time points post injury, (#) represent a significant difference between an experimental group and the naïve group at the same time point post injury, (▲) represent a significant difference between an experimental group and another experimental group at the same time point post injury. At 5 dpi: 4 animals for naive, 4 animals for rSham, 4 animals for rmTBI, 4 animals for cranio and 4 animals for CCI. At 35 dpi: 6 animals for naive, 4 animals for rSham, animals for rmTBI, 5 animals for cranio and 4 animals for CCI.

Figure S7

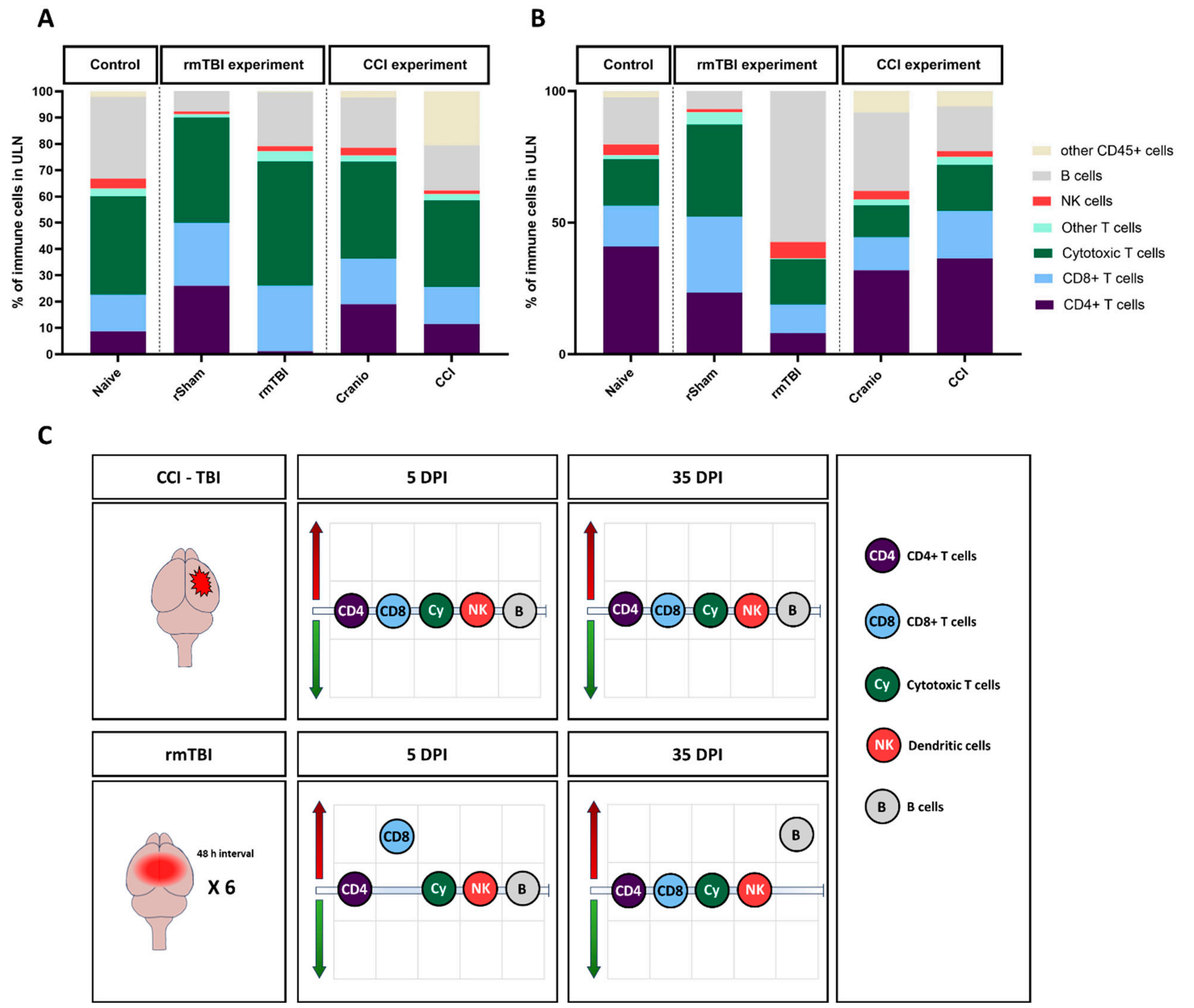

Figure S7: Upper lymph nodes analysis from the rmTBI and CCI experiments. Percentages of immune cells in ULN tissue (A) at 5 dpi and (B) at 35 dpi. (C) Summary of observed changes in the two models in ULN samples. Red arrow indicates increase and green arrow indicates decrease.

Table S4

Table S4: Summary of all the observed changes in immune cells in the two models in upper (axillary and cervical) lymph nodes sample when compared to the naïve group. (↑S): significant increase, (↓S): significant decrease, (-): non-significant difference.

Cell type	rSham		rmTBI		Cranio		CCI	
	Acute phase	Subacute phase	Acute phase	Subacute phase	Acute phase	Subacute phase	Acute phase	Subacute phase
**Total T cells**	-	-	s*	↓S, s*	-	-	-	-
**CD4+ T cells**	-	-	-	-	-	-	-	-
**CD8+ T cells**	↑S	-	↑S	-	-	-	-	-
**Cytotoxic T cells**	-	-	-	-	-	-	-	-
**NK cells**	-	-	s*	s*	-	-	-	-
**B cells**	-	-	s*	↑S, s*	-	-	-	-

*** Represent a significant difference between an injured group and its sham group at the same time point post injury.**

Immune system response in lower lymph node (LLN)

Figure S8

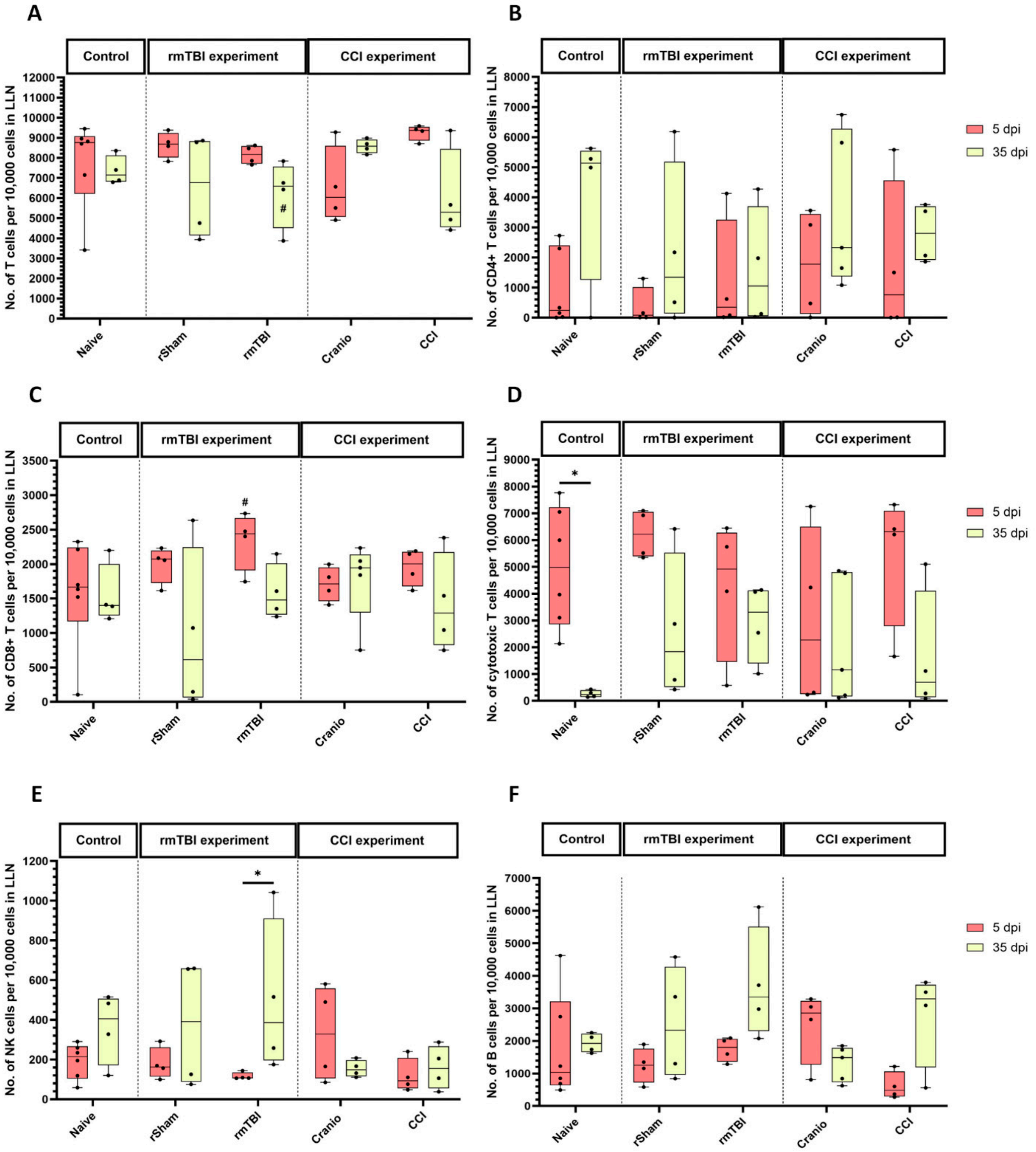

Figure S8: Inguinal lymph nodes sample analysis from the rmTBI and CCI experiments. (A) Absolute count of T cells in lower lymph nodes (LLN) per 10,000 gated cells, (B) Absolute count of CD4+ helper T cells in the LLN per 10,000 gated cells, (C) Absolute count of CD8+ T cells in the LLN per 10,000 gated cells, (D) Absolute count of cytotoxic T cells in the LLN per 10,000 gated cells, (E) Absolute count of NK cells in the LLN per 10,000 gated cells, (F) Absolute count of B cells in the ULN per 10,000 gated cells. Results represent the median, minimum and maximum. Data were analysed using the Friedman's test (two-way analysis of variance by ranks) followed by the Dunn post-hoc test; *P<0.05, $^{\#}$P<0.05. (*) represent a significant difference within an experimental group at different time points post injury, (#) represent a significant difference between an experimental group and the naïve group at the same time point post injury. At 5 dpi: 4 animals for naive, 4 animals for rSham, 4 animals for rmTBI, 4 animals for cranio and 4 animals for CCI. At 35 dpi: 6 animals for naive, 4 animals for rSham, animals for rmTBI, 5 animals for cranio and 4 animals for CCI.

Figure S9

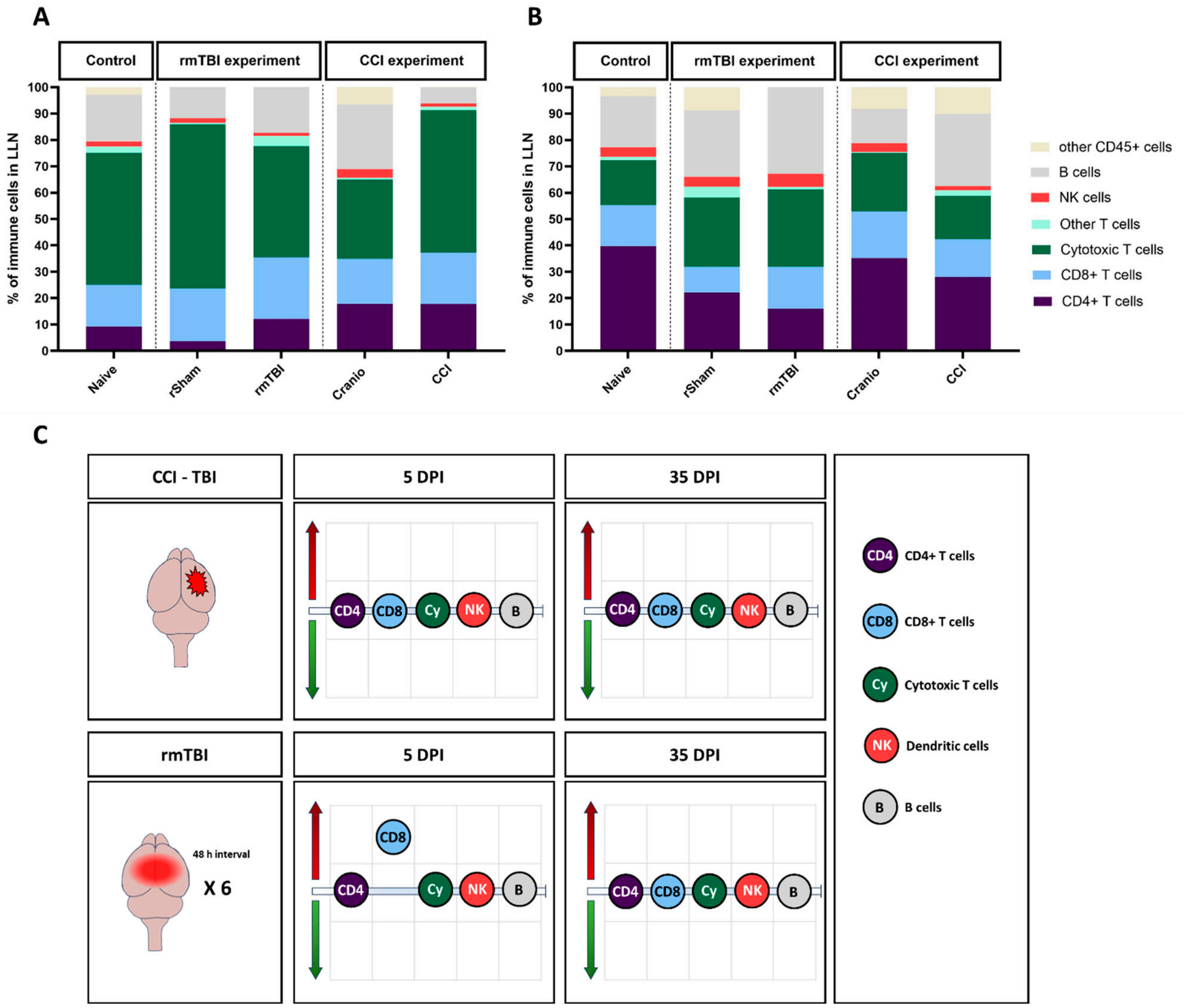

Figure S9: Lower lymph nodes analysis from the rmTBI and CCI experiments. Percentages of immune cells in LLN tissue (A) at 5 dpi and (B) at 35 dpi. (C) Summary of observed changes in the two models in LLN samples. Red arrow indicates increase and green arrow indicates decrease.

Table S5

Table S5: Summary of all the observed changes in immune cells in the two models in lower (inguinal) lymph nodes sample when compared to the naïve group. (↑S): significant increase, (-): non-significant difference.

Cell type	rSham		rmTBI		Cranio		CCI	
	Acute phase	Subacute phase	Acute phase	Subacute phase	Acute phase	Subacute phase	Acute phase	Subacute phase
**Total T cells**	-	-	-	-	-	-	-	-
**CD4+ T cells**	-	-	-	-	-	-	-	-
**CD8+ T cells**	-	-	↑S	-	-	-	-	-
**Cytotoxic T cells**	-	-	-	-	-	-	-	-
**NK cells**	-	-	-	-	-	-	-	-
**B cells**	-	-	-	-	-	-	-	-
